# Diversity and Dispersal of Fungi Along a Subtropical Land‐to‐Sea Continuum

**DOI:** 10.1111/1758-2229.70297

**Published:** 2026-04-03

**Authors:** M. Meyneng, L. Tedersoo, G. Burgaud, V. Mikryukov, F. Carriconde, H. Lemonnier, R. Siano

**Affiliations:** ^1^ Ifremer DYNECO, BP70 Plouzané France; ^2^ Institute of Ecology and Earth Sciences University of Tartu Tartu Estonia; ^3^ Univ Brest INRAE, Laboratoire Universitaire de Biodiversité et Écologie Microbienne Plouzané France; ^4^ Institut Universitaire de France France; ^5^ IAC Nouméa New Caledonia; ^6^ Ifremer, CNRS, IRD, Univ Nouvelle‐Calédonie Univ La Réunion, ENTROPIE Nouméa Nouvelle‐Calédonie France; ^7^ MARBEC, Univ. Montpellier CNRS, Ifremer, IRD Sète France

**Keywords:** coalescence, coastal mycobiome, dispersal, fungal bioindicators, land‐to‐sea continuum

## Abstract

This study examines the fungal and fungal‐like stramenopile communities along the land‐to‐sea continuum to explore microbial connectivity across terrestrial and marine ecosystems. In addition to providing new ecological knowledge on coastal communities, the objectives were to assess the presence of terrestrial fungi and evaluate their potential as bioindicators of riverine influence on marine environments. Conducted in New Caledonia (southwest Pacific), the research involved 113 marine samples (water and sediment) and 148 additional soil samples from tropical/subtropical Pacific regions. Sampling spanned spatial and temporal gradients, including dry periods and a post‐cyclone runoff event. We used high‐throughput long‐read amplicon sequencing of the full‐length ITS rRNA gene, complemented by inference of fungal ecological traits from genus‐level annotations. In total, 1653 fungal OTUs were identified in marine samples, with distinct communities in sediment and water. Terrestrial fungal taxa were also detected in marine environments, with 306 genera exhibiting varying occurrence across sample types and periods. The cyclone runoff temporarily increased the richness of terrestrial fungal taxa by up to threefold, underscoring the impact of extreme events on marine coastal assemblages. Overall, this work provides the first study of fungal communities along New Caledonia's coastlines, highlighting fungal dispersal as a key component of land‐to‐sea connectivity.

## Graphical abstract

This study investigates the marine mycobiome by integrating cross‐habitat sampling with a long‐read amplicon sequencing approach. The results provide novel insights into the largely unexplored fungal diversity in coastal ecosystems and highlight the role of dispersal, evidenced by the detection of transported terrestrial fungal taxa.

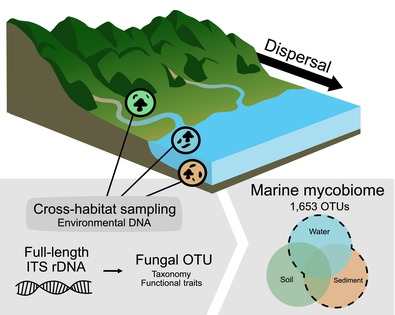

## 1 Introduction

The concept of hydrological connectivity states that water is a vector of matter, energy and organisms across different environments (Bracken et al. 2013; Freeman et al. 2007). Riverine waters, in particular, play key roles in facilitating this connectivity along the land‐to‐sea continuum, transporting nutrients, organic matter and (micro)organisms from upstream into coastal zones (Frigstad et al. 2020; LeBrun et al. 2018; Xenopoulos et al. 2017). Understanding these cross‐ecosystem exchanges is essential for elucidating the ecological processes, biogeographical and biodiversity patterns of coastal environments. Beyond the continuous influence of riverine flows, these land‐to‐sea fluxes can also be shaped by episodic but intense disturbances. In particular, extreme weather events such as cyclones and intense rainfall increase land‐sea connectivity (Meyneng, Lemonnier, et al. 2024). These events are projected to become more frequent and intense under climate change, especially in tropical regions, thereby amplifying short‐term but significant pulses of hydrological connectivity that might have important cascading effects (e.g., Seneviratne et al. 2021).

Among the elements transported by rivers, numerous microorganisms are carried along this continuum, ultimately discharging into coastal areas far from their native habitats. This transport of organisms and their introduction into non‐native environments can result in community coalescence, that is the mixing of entire microbial assemblages (Rillig et al. 2015), which may alter local biodiversity, community structure, biogeochemical processes, ecological functioning and evolutionary trajectories (Chang et al. 2024; Custer et al. 2022; Rillig et al. 2015). The presence of freshwater and terrestrial organisms, especially microorganisms, in marine environments has been documented in different coastal regions. Transported taxa, often termed ‘tourists’ due to their temporary presence in non‐native environments (Snell Taylor et al. 2018), may not actively grow in their new environment and may be considered as biological noise (Amend et al. 2019; Nadal et al. 2022; Snell Taylor et al. 2018), but nonetheless reflect hydrological connectivity. For instance, studies on bacterial communities sampled across distinct environmental compartments have emphasised the tracing of source‐sink dynamics (Hauptmann et al. 2016) and the importance of lifestyle strategies (e.g., particle‐associated vs. free‐living bacteria; Pan et al. 2022).

Fungi and fungal‐like stramenopiles (comprising Oomycota, Hyphochytriomycota and Labyrinthulidia) remain so far understudied in this context and offer a promising avenue to explore microbial dispersal across ecosystems. Although early studies have explored their ecological importance (e.g., in biogeochemical processes as saprotrophs; Burgaud et al. 2022) and global diversity in open‐water environments (Hassett et al. 2020), the factors driving their diversity and community structure across the land‐to‐sea continuum are still largely unknown. Some fungal species are ubiquitous and can thrive in either terrestrial or aquatic environments, demonstrating wide environmental tolerance (Burgaud et al. 2013). Such species are referred to ‘partially aquatic’ or ‘facultative marine fungi’, such as *Aspergillus flavus* (Ramírez‐Camejo et al. 2012). However, others are strictly terrestrial (e.g., plant‐associated fungi), and their presence may serve as indicators of passive water‐driven transport (Banchi et al. 2024). Because certain terrestrial fungi exhibit salinity tolerance (Raghukumar 2017) and effective dispersal strategies (e.g., sporulation), we hypothesise that their detection in marine systems may reflect riverine inputs and changes in coastal fungal community composition.

Here, we characterised fungal communities across soil, sediment, and surface water samples collected in New Caledonia, a tropical archipelago in the southwest Pacific. Our dataset includes 113 local environmental samples, with marine water collected across dry seasons and after a cyclone‐induced runoff event (Meyneng, Lemonnier, et al. 2024). In addition, we analysed 148 additional soil samples from Pacific islands to broaden the biogeographic context, that is, the degree of biodiversity overlap and exchange among terrestrial and marine ecosystems. Knowing that analyses of fungal diversity highly depend on the chosen genetic marker (Amend et al. 2019; Nilsson et al. 2019), and given that our aim was to assess the best taxonomic resolution for differentiating terrigenous organisms from their marine congeners, we chose to use sequencing of the full‐ITS amplicon of the rRNA gene, considered the most effective marker for barcoding fungal species (Tedersoo et al. 2022), despite certain limitations discussed in Kauserud (2023). We hypothesised that integrating this long‐read sequencing approach with cross‐ecosystem datasets would enable the detection of terrestrial fungal taxa that act as indicators of riverine inputs. This exploratory work aims (1) to provide the first description of fungal communities in New Caledonia's marine coastal ecosystems, (2) to assess the presence and spatiotemporal dynamics of terrestrial fungi in marine habitats, and (3) to study the potential of specific fungal taxa as bioindicators of terrestrial inputs in marine settings. By doing so, this work provides the first study of diversity and dispersal of fungal communities along a tropical land‐to‐sea continuum located in the southern hemisphere, contributing to a broader understanding of the role of fungi in coastal ecosystem dynamics and environmental monitoring.

## 2 Methods

### 2.1 Sampling Strategy

**FIGURE 1:**
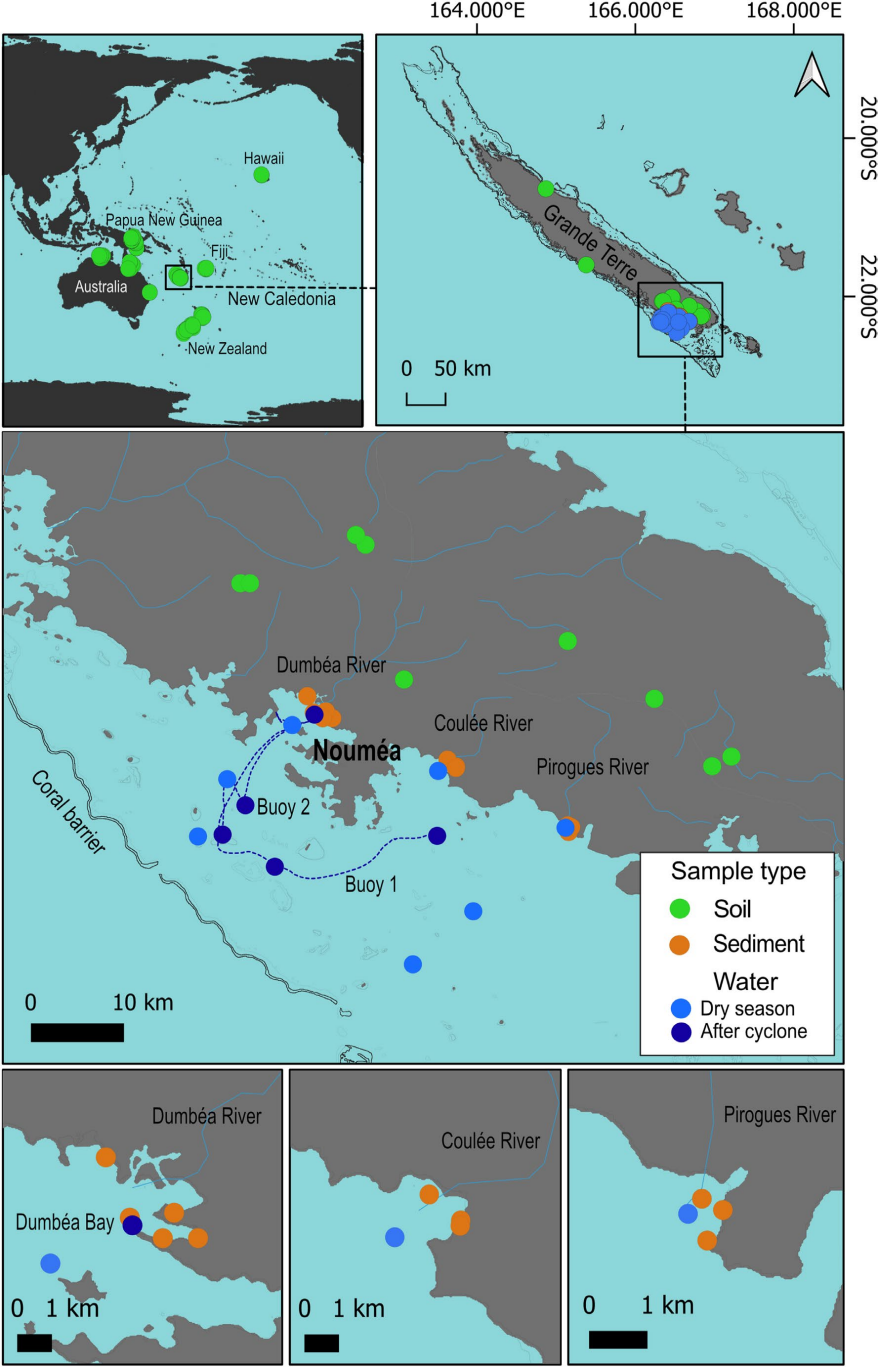
Sampling design across terrestrial and marine ecosystems in New Caledonia (water samples collected during the dry season were classified into three categories along a coast‐to‐offshore gradient: Coastal, intermediate (inter), and offshore (off) stations).

Most samples were collected in New Caledonia, a French territory located in the southwest Pacific Ocean. Marine samples (*n* = 113, including surface water and sediment) were obtained from the southwest area of the main island, called ‘The Grande Terre’, near the urban area of Nouméa city (Figure 1). Surface water samples (0–2 m, *n* = 80, Meyneng, Lemonnier, et al. 2024) were collected across three sampling campaigns: two during the dry season (September 2019 and December 2020) and one immediately following an extreme weather event, that is the passage of Uesi cyclone in February 2020. In New Caledonia, rivers and lagoons are subject to extreme weather events during the rainy season, lasting from a few hours to several days, leading to rapid transfer of material resulting from soil erosion. After the cyclone, Dumbéa Bay (DB) was sampled daily over six consecutive days. The Dumbéa River plume was tracked using two drifting buoys released from the bay, with daily sampling carried out for two consecutive days following their release. Water samples were filtered sequentially into three size fractions (> 20 μm, 20–3 μm and 3–0.2 μm) to assess size‐related community structure using sterile polycarbonate filters. Surface sediments (0–5 cm, *n* = 33, Meyneng, Siano, et al. 2024) were collected at low tide in March 2022 using PVC cores deployed near the mouths of the Dumbéa (DB), Coulée (Co) and Pirogues (Pi) rivers. All samples were stored at −20°C until DNA extraction to preserve microbial communities and prevent degradation.

Soil samples from New Caledonia were mainly collected in the southern province from the peridotite massif, with the exception of one sample from the Northern Province (Figure 1). Following a standardised protocol, these soil samples (*n* = 18, Tedersoo et al. 2021) were collected using cores at 5 cm depth in December 2014 and March 2015. Other soil samples collected in the context of the Global Soil Mycobiome consortium survey (Tedersoo et al. 2021) were also considered in this study (including 7 soil samples from Fiji, 34 from New Zealand, 4 from Hawaii, 34 from Australia and 69 from Papua New Guinea), spanning various terrestrial ecosystems. Detailed sampling protocols for each sample type are provided in the respective works (Meyneng, Lemonnier, et al. 2024; Meyneng, Siano, et al. 2024; Tedersoo et al. 2021).

### 2.2 DNA Extraction and Amplification

DNA extraction was performed using different kits adapted to each substrate: PowerMax Soil DNA Isolation kit (Qiagen) for soil and sediment, and Nucleospin Plant II Mini kit (Macherey–Nagel, Hoerdt, France) for water samples, following manufacturers' protocols. Amplification targeted the full‐length ITS region of the rRNA gene using the eukaryote‐specific primers ITS9mun (5′‐GTA CAC ACC GCC CGT CG‐3′) and ITS4ngsUni (5′‐CCT SCS CTT ANT DAT ATG C‐3′) (Tedersoo and Anslan 2019; Tedersoo and Lindahl 2016). PCR amplification followed the protocol of Tedersoo et al. (2021). Negative controls (no‐template controls) were included in each PCR batch to monitor for contamination. PCR products were checked on 1% agarose gel electrophoresis. PCR products were pooled for library preparation based on DNA concentration (between 1 and 20 μL), purified using FavorPrep GEL/PCR Purification DNA fragments Mini Kit (Favorgen) and sequenced using the PacBio (Pacific Biosciences) platform at the Norwegian Sequencing Centre (University of Oslo).

### 2.3 Bioinformatic Analysis

#### 2.3.1 OTU Clustering

Sequencing reads were processed using the NextITS pipeline v.0.6.0 (Mikryukov et al. 2024; https://github.com/vmikk/NextITS), optimised for full‐length ITS metabarcoding. Demultiplexing was performed with LIMA v.2.9.0 (PacBio) with a minimum barcode score of 93. Full‐length ITS regions were extracted using ITSx v.1.1.3 (Bengtsson‐Palme et al. 2013) to minimise biases associated with including SSU and LSU regions (Lindahl et al. 2013; Tedersoo et al. 2022). Prior to clustering, we used the UNOISE algorithm (Edgar 2016) to denoise reads and remove sequencing errors. Given that ASV resolution does not always provide the most accurate representation of fungal diversity (Kauserud 2023; Tedersoo et al. 2022), we clustered denoised reads into OTUs using VSEARCH v.2.27.0 (Rognes et al. 2016) with a similarity threshold of 98%. This threshold was used as a global compromise to balance taxon lumping (when interspecific divergence is low) against taxon splitting (when intraspecific ITS variation and residual errors inflate diversity). Although optimal thresholds could vary among lineages, implementing lineage‐specific clustering is currently not feasible. Post‐clustering curation was conducted using the LULU algorithm (Frøslev et al. 2017) to mitigate the inclusion of spurious OTUs, applying a minimum similarity threshold of 95% and relative co‐occurrence of 0.95.

#### 2.3.2 Taxonomic Assignment

Taxonomic classification was performed by querying the sequences against the Eukaryome database v.1.7 (Tedersoo et al. 2024) using BLASTn v2.16.0 (Camacho et al. 2009). For each OTU, the top matches were retained. To reduce the likelihood of incorrect taxonomic assignments, several conservative thresholds were set for taxonomic confidence: Kingdom‐level assignment required a minimum E‐value of e‐50, Phylum e‐55, Class e‐70 and Order e‐80. For the genus and species level, we used variable similarity thresholds depending on the phylum because of the non‐homogeneous evolutionary rate of the ITS region among different fungal groups (Vu et al. 2022). Only genus assignments with > 95% sequence similarity for Ascomycota, > 90% for Basidiomycota and > 80% for other phyla were retained. The same approach was applied for species assignment using > 99.5% for Ascomycota, > 98% for Basidiomycota and > 95% for other phyla. For subsequent analyses, only OTUs classified as fungi and fungal‐like stramenopiles (Oomycota, Hyphochytriomycota and Labyrinthulidia) were retained, given the expected dominance of the latter in marine environments. In addition, reads associated with a few specific taxonomic groups (e.g., diatoms) were also retained in order to explore potential correlations.

#### 2.3.3 Functional Annotation

In order to better assess the origin and ecology of fungi from a functional perspective, we used the FungalTraits database v.1.2 (Põlme et al. 2020) to annotate our taxa at the genus level (Data S3). The ‘primary_lifestyle’ trait was used to consider the common natural habitat of fungal taxa. The ‘Aquatic_habitat_template’ trait was used to distinguish partially or obligately aquatic, freshwater, or marine environment‐associated fungi from non‐aquatic fungi. Compared to the original dataset, the category ‘partly_aquatic’ grouped all original traits related to partial aquatic affiliation, including ‘partly_freshwater’, ‘partly_marine’ and ‘partly_non_aquatic’. For OTUs assigned at the species level, additional research using available datasets was conducted to improve habitat annotation when possible. The datasets used were primarily GlobalFungi (Větrovský et al. 2020), MarineFungi (Jones et al. 2019) and FreshwaterFungi (Calabon et al. 2020). These functional traits reflect current knowledge of fungal genera; however, they should be interpreted with caution given the high versatility of some taxa and the many unresolved aspects of fungal ecology.

### 2.4 Statistical Analyses

All statistical analyses were conducted in R software v 4.3.3 (R Core Team 2024). The sample completeness was quantified using the coverage estimator of Chao and Jost (2012), applying the singleton‐corrected formulation to reduce small‐sample bias from rare sequence variants as implemented in the metagMisc package v.0.6.0 (Mikryukov 2025). Before analyses, singletons and short‐sequence OTUs (< 250 bp) were removed, and CSS normalised the abundance of reads (Paulson et al. 2013). The variability of fungal communities was assessed across all soil, sediment and water samples at different spatial and temporal scales. Within the eukaryotic dataset, fungal relative abundance (% of reads) was used as a proxy for fungal contribution, whereas community richness was assessed using the number of fungal OTUs and Shannon index. Differences in average fungal contribution and richness among substrates were tested using Kruskal‐Wallis and Dunn tests using ‘rstatix’ package (Kassambara 2019). Taxonomic composition was analysed at both phylum and genus levels. Beta‐diversity patterns in water and sediment samples were evaluated using principal coordinate analysis (PCoA) based on Jaccard dissimilarity matrices at multiple taxonomic resolutions. Statistical significance of community differences between sample types and temporal groups was tested using permutational multivariate analysis of variance (PERMANOVA; M. J. Anderson 2017) with 999 permutations using the ‘vegan’ package (Oksanen et al. 2022). Pairwise comparisons were conducted when overall tests were significant. The amount of specific and shared OTUs among substrates was visualised using the ‘UpsetR’ package (Conway et al. 2017). Functional traits were incorporated to infer putative ecological characteristics underlying the observed spatiotemporal patterns (e.g., aquatic vs. terrestrial habitat). Surface marine water salinity, temperature and chlorophyll *a* (Chl *a*) concentration were also associated with fungal diversity data to evaluate the influence of environmental changes on fungal community dynamics, especially after the cyclone.

## 3 Results

The raw dataset comprised 1,008,592 reads and 312,457 OTUs, with variable values per sample depending on the substrate (average of 3628 ± 2411 reads and 703 ± 372 OTUs per sample; Figure S1). Median achieved sample coverage was 0.939 (IQR 0.928–0.967; with median sequencing depth of 3025; Data S2), indicating that a high proportion of each community's sequencing reads belonged to detected taxa and that the sampled communities were captured well. The mean sequence length was 581 bp (minimum length: 36 bp; maximum length: 2776 bp, Figure S2). Singletons (268,175 OTUs) and OTUs with sequences shorter than 250 bp (679 OTUs) were excluded. Then, for further analyses, only fungi and fungal‐like stramenopiles (Oomycota, Hyphochytriomycota and Labyrinthulidia) were retained, while protists (17,869 OTUs), metazoans (4097), plants (433) or unidentified sequences (1859) were removed. This filtering resulted in a refined dataset of 406,751 fungal sequences across 278 samples, representing a final 19,932 OTUs, including 1653 OTUs present in marine samples. The functional database allowed annotation of 9575 OTUs, including 607 of the 1653 OTUs present in marine samples. The remaining OTUs were either not assigned at the genus level (10,016 OTUs) or were assigned to genera not annotated in the database used (341 OTUs assigned to 88 different genera).

### 3.1 Distinct Fungal Communities

**FIGURE 2:**
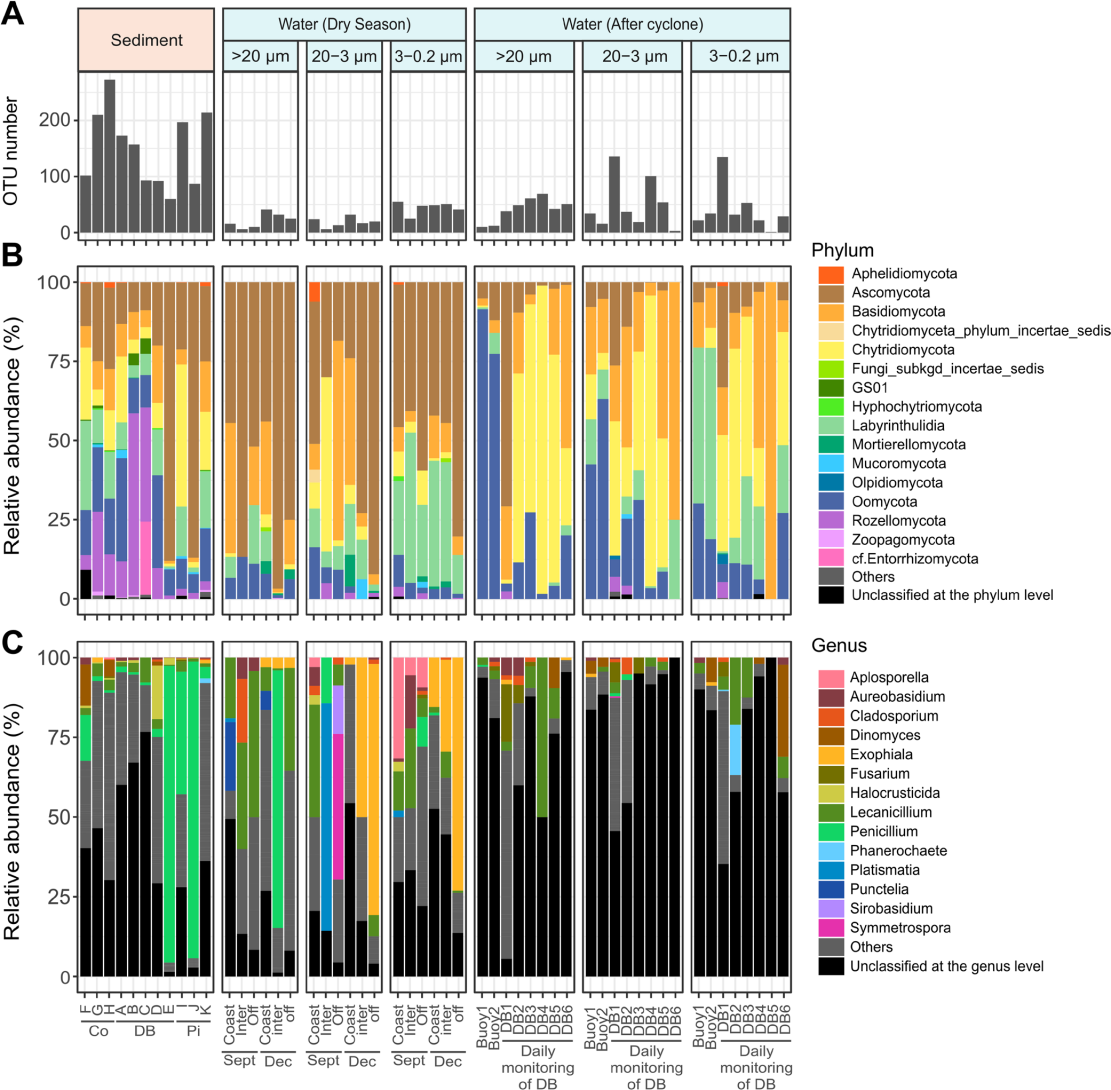
Variation of (A) richness and taxonomic composition, described at (B) the phylum and (C) genus level, in sediment and water samples. The ‘Others’ category (in grey) gathered phyla showing less than 1% (6 phyla) and genera showing less than 15% in all samples (337 genera). (Co: coulée, DB: dumbéa; Pi: pirogues; Inter: intermediary; Off: offshore; DB: dumbéa bay).

Fungal communities varied markedly across environmental substrates, in terms of contribution (% of OTUs and reads among microeukaryotes), structure and composition (number and relative abundance of OTUs) and lifestyle (functional assignment associated with taxonomic composition). Fungi dominated soil samples among all eukaryotes, with OTUs representing 70% ± 11% of total OTUs, compared to only 8.1% ± 3.4% in sediment and 6.2% ± 12% in water (Kruskal‐Wallis, *p* value < 0.01; Figure S3). A similar pattern was observed for read proportions (77% ± 15%, 7.3% ± 13% and 3.0% ± 11%, respectively) (Kruskal‐Wallis, *p* value < 0.01; Figure S3). Focusing on the fungal community (fungi and fungal‐like stramenopiles) of New Caledonia, strong richness differences were also found, with soil samples having a higher mean number of fungal OTUs per sample (343 ± 128) compared to sediment and water samples (60 ± 47 and 21 ± 22, respectively) (Kruskal‐Wallis, *p* value < 0.01; Figure 2A, Figure S4).

Beyond richness, the taxonomic composition of fungal communities was also clearly distinct among substrates, at both phylum and genus levels (Figure 2B,C and Figure S5). PERMANOVA revealed significant differences in fungal community composition among substrates, although the proportion of explained variance was low (*R* ^2^ = 0.031, *F* = 4.37, *p* < 0.001), with pairwise comparisons showing significant differences between all substrate pairs (*R* ^2^: sediment vs. water = 0.027, sediment vs. soil = 0.017, water vs. soil = 0.023; all *p* < 0.01). Soil samples from different geographic regions showed a certain spatial variability (Figure S6) but were dominated by Ascomycota (42% ± 7.8% of reads) and Basidiomycota (33% ± 7.6%), followed by variable contributions from Zoopagomycota (4.7% ± 5.3%), Mortierellomycota (8.8% ± 8.5%) and Mucoromycota (4.2% ± 4.3%). Marine samples, by contrast, showed a higher proportion of Labyrinthulidia (11% ± 8.1% in sediment, 13% ± 15% in water and 0.01% ± 0.01% in soil samples) and Oomycota (16% ± 8.6%, 15% ± 20% and 0.4% ± 0.5%, respectively) (Figure 2B). Furthermore, sediment showed a different composition than water samples, visible at the phylum level by the higher relative abundance of Rozellomycota (15% ± 18% in sediment against only 0.7% ± 1.4% in water samples).

At the genus level, some taxa dominated specific sample types or time points (Figure 2C). For example, *Penicillium* accounted for over 80% of reads in several sediment and water samples, while *Exophiala* reached > 50% of reads in December water samples. *Symmetrospora* was abundant in offshore water in September (46%). More ubiquitous genera included *Lecanicillium* (detected in all of the 24 sample groups) and *Aureobasidium* (present in 62%). Still, a significant proportion of reads could not be assigned at the genus level (representing an average of 50% ± 32% of reads in all sample groups).

Beyond the differences in communities observed between substrates, variability on a finer scale was also observed. Among these patterns, surface water communities varied between the two dry seasons, which were mainly separated by water temperature (mean of 22.3°C ± 0.11°C and 25.8°C ± 0.33°C in September and December, respectively; Table S1). Differences in composition were especially visible at the genus level (e.g., dominance of *Exophiala* in December, Figure 2C), despite stations being located at the same sites. Similarly, sediment samples showed an inter‐station variability, which was highly visible at the Pirogues site both in terms of richness and composition, reflecting the range of measured parameters within a station (mean of 29.9 ± 4.04 of salinity and 2.51 ± 0.9 of Chl a (g g^−1^ of wet sediment); Table S1). Finally, regarding the methodological approach, variability within size classes was evident, with greater differences observed for the > 20 μm filtration, while there was a high degree of similarity in taxonomic composition between the 20–3 μm and 3–0.2 μm size fractions (Figure 2B,C). This variability among size fractions was not visible in terms of richness (Figure S7).

### 3.2 Fungal Functional Diversity

**FIGURE 3:**
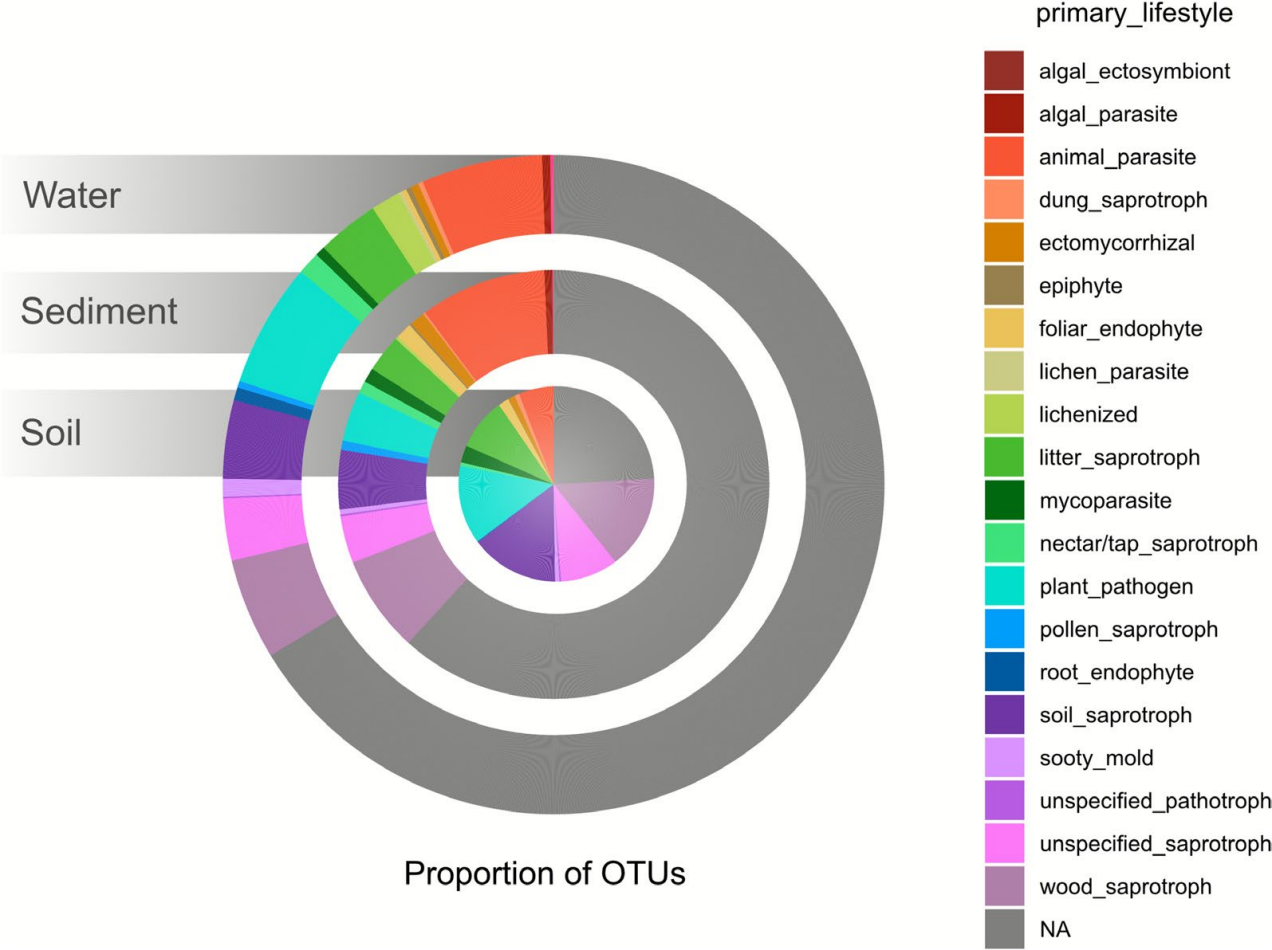
Proportion of primary lifestyle traits among fungal OTUs in each substrate.

Functional annotation based on genus‐level assignment provided insights into fungal lifestyles (Figure 3, Data S3). About half of the OTUs (52% of the 19,932 OTUs) could not be annotated functionally. As mentioned in Põlme et al. 2020, such functional annotations are highly affected by the still unknown ecology of many genera and species. Furthermore, the ecological versatility of many fungi, that is the fact that many fungi exhibit ecological plasticity and perform multiple roles, further limits the ability to draw definitive conclusions, and observed patterns should therefore be interpreted as indicative rather than as strict ecological assignments. Still, a high proportion of OTUs were annotated in soil samples (76%) and showed a dominance of OTUs associated with soil, wood and plants (wood saprotroph: 15.3%, soil saprotroph: 14.9%, plant pathogen: 13.4%, and litter saprotroph: 8.77%). Sediment and water samples showed a lower percentage of annotated OTUs (38% in sediment and 34% in water) and revealed the presence of algae‐associated fungi (algal ectosymbionts: 0.08% in sediment, and algal parasites: 0.54% in sediment and 0.4% in water). Sediment samples also showed a higher proportion of fungi annotated as animal parasites (9.52%, compared to 5.78% and 5.92% in soil and surface water, respectively).

### 3.3 Terrestrial Fungi in Marine Areas

**FIGURE 4:**
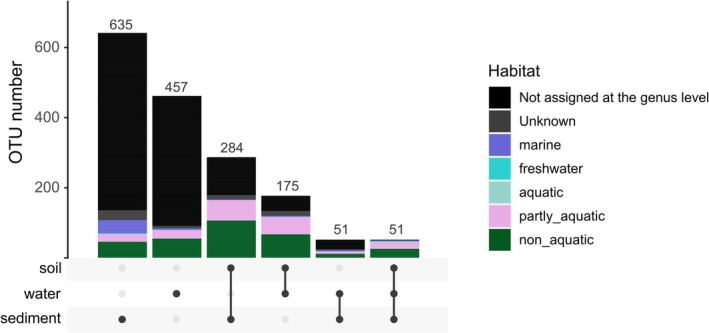
Number of total and shared fungal OTUs among the different substrates (excluding the 18,279 OTUs specific to soil samples), associated with their inferred habitat.

Among the 19,932 fungal OTUs identified across 278 samples, the majority (18,279 OTUs, representing 91.7% of all fungal OTUs) were specific to soil samples. For marine samples, 1653 fungal OTUs were detected in at least one marine sample, either sediment or surface water. Of these, 635 and 457 OTUs were specific to sediment and water, respectively, while 51 OTUs were shared across all three sample types (soil, sediment and water) (Figure 4). The functional annotation revealed that a subset of these shared OTUs corresponded to taxa typically associated with terrestrial environments, as they are known to have a non‐aquatic habitat. Such non‐aquatic taxa represented 37% of OTUs shared between soil and sediment, 38% between soil and water, 22% between water and sediment and 49% of those present across all three substrates.

### 3.4 Variability After a Cyclone‐Induced Runoff

**FIGURE 5:**
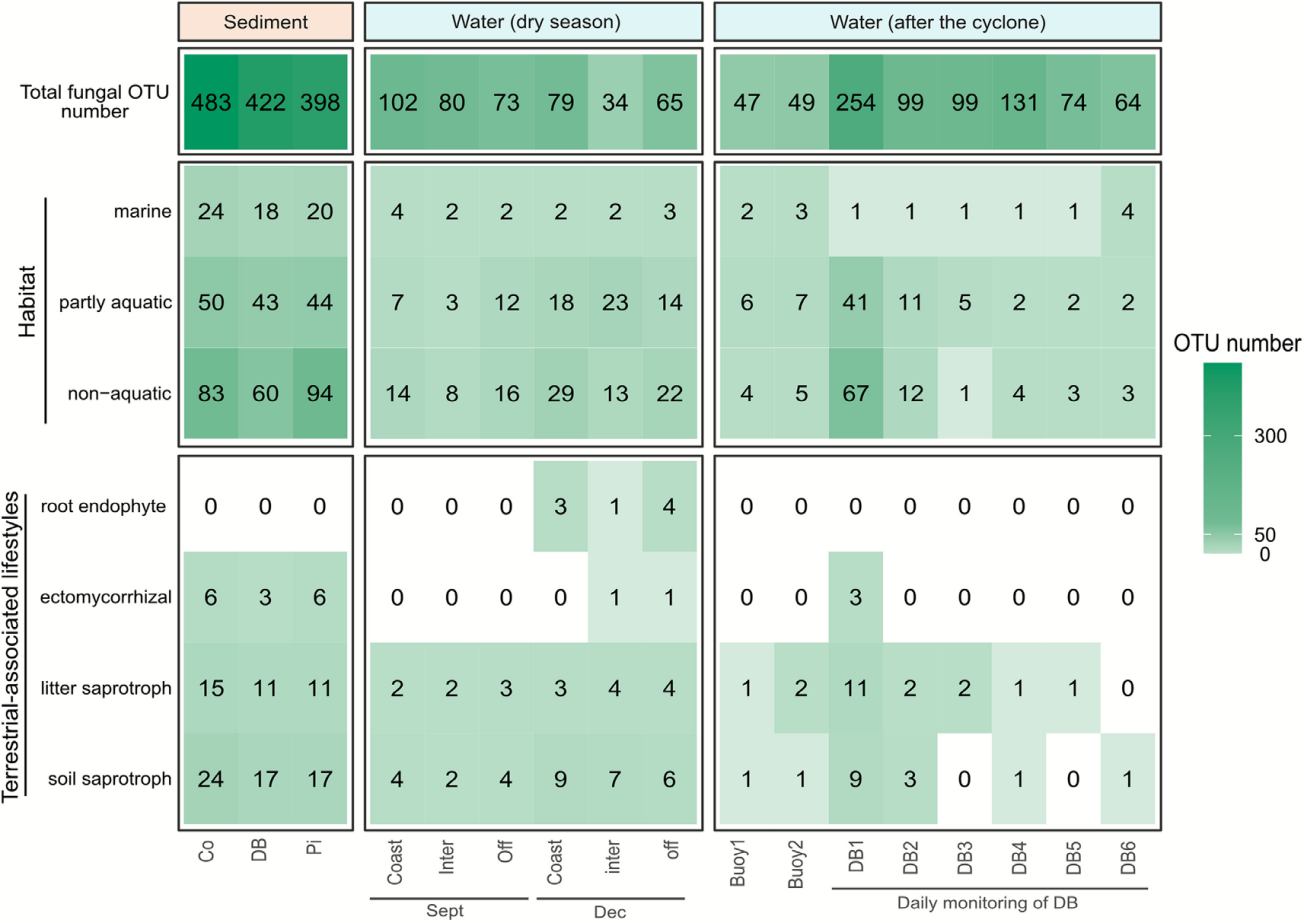
Number of fungal OTUs in the marine samples with some functional categories of habitat and primary lifestyle (without distinction of filter size).

Marine fungal communities varied depending on the time period. A significant difference in both composition and structure was observed in surface waters when comparing dry season water samples (September 2019 and December 2020) to water samples immediately following cyclone Uesi (February 2020) (Table S1, Figures 2 and 5 and Figures S5, S7 and S8). PERMANOVA confirmed significant differences in fungal community composition between dry season and post‐cyclone samples (*R* ^2^ = 0.080, *F* = 3.30, *p* < 0.001). For instance, coastal water samples collected on the first day after the cyclone harboured 254 fungal OTUs, clearly more than those observed during dry‐season samples in September 2019 (79 OTUs) and December 2020 (102 OTUs). This peak in richness appeared consistent with a sharp drop in salinity to a value of 2.2 (Table S1), reflecting the massive increase in Dumbéa river flow induced by the cyclone event (1188 m^3^ s^−1^ compared to an annual mean of 3 m^3^ s^−1^). The 6 days of monitoring of Dumbéa Bay showed that after this initial increase, the number of fungal taxa reached values closer to dry season samples (from 99 OTUs on Day 2 to 64 OTUs on Day 6, with a peak of 131 on Day 4) and salinity went back to usual value on day 2 (35.9; Table S1). In these water samples, some phyla showed a higher proportion after the cyclone compared to the dry season, such as Chytridiomycota (5.9% ± 13% in the dry season and 32% ± 30% after the cyclone) and Oomycota (5.8% ± 4.8% and 22% ± 25%) (Figure 2B).

The run‐off induced by the cyclone provoked changes in the functional composition of the fungal communities. Among the OTUs detected post‐cyclone, 67 were assigned to non‐aquatic fungal taxa. Some OTUs only appeared during the post‐cyclonic monitoring, such as representatives of *Dinomyces* sp., a known algal parasite, which increased in read proportion from days 4 to 6 (+27%), concordant with a marked increase in chlorophyll *a* concentration (> 1.5 μg L^−1^) (Figure 2C, Table S1). Similarly, the read abundance of Chytridiomycota showed a significant positive correlation with diatom read abundance (Figure S9), suggesting potential parasitic interactions. Conversely, several OTUs persisted only for a few days, such as *Phytophthora hydropathica*, a fungus‐like plant pathogen, which was detected only on Days 1 and 2 after the cyclone. The increase in terrestrial OTUs in marine waters, which may be underestimated because of incomplete functional annotations, was transient and did not lead to a significant change at the scale of the 6‐day monitoring period (Figure S8). Along the river plume tracked by the drifting buoys that followed the Dumbéa River plume, seven non‐aquatic OTUs were detected, including *Coprophilum lecanicillium*, *Concrescens rigidoporus*, *Penicillium janczewskii*, *Pseudopithomyces palmicola*, *Porostereum* sp., *Phlebiopsis* sp. and *Curvularia* sp. Additionally, 15 ectomycorrhizal and six root‐endophytic OTUs were recorded across all marine samples, with three ectomycorrhizal taxa (*Pisolithus albus*, *Rhizopogon villosulus and Inocybe* sp.) detected exclusively post‐cyclone (Figure 5).

## 4 Discussion

### 4.1 Fungal Communities Across Habitats

Our work provides the first comprehensive description of fungal communities (fungi and fungal‐like stramenopiles) along the land‐to‐sea continuum in New Caledonia. The use of full‐length ITS PacBio amplicon sequencing improved taxonomic resolution of marine fungal communities compared to short‐read ITS2 Illumina sequencing, which underrepresented early‐diverging fungal lineages (see Appendix A).

Fungal dominance was evident in soil samples, where fungi represented on average 77% ± 15% of all eukaryotic OTUs and 70% ± 11% of all eukaryotic reads (Figure S3). In contrast, despite some studies showing that fungal organisms could be dominant in some aquatic areas, reaching 50% of reads in freshwater environments (Jones et al. 2014), our marine samples showed much lower fungal contribution with an average of mainly < 10% of fungal reads (7.3% ± 13% in sediment and 3.0% ± 11% in water samples, Figure S3), closer to the finding of Comeau et al. (2016). Fungal contributions were low in our dataset, particularly in surface waters, which is consistent with other observations in oceanic systems. Recent global surveys similarly report modest fungal representation in marine environments. For instance, the Tara Oceans expedition found fungal taxa accounting for only ~0.7% of micro‐eukaryotic reads and OTUs across 334 photic‐zone plankton samples (de Vargas et al. 2015). Hassett et al. (2020) reported fungi representing 7.8% of eukaryotic sequences in a large‐scale meta‐analysis of marine shotgun sequencing datasets. In contrast, fungal relative abundance is significantly higher in freshwater systems: fungi comprised ~25% of micro‐eukaryotic OTUs in lakes and rivers (Debroas et al. 2017) and up to 30% of OTUs in rRNA‐based datasets targeting active fungi (Lepère et al. 2019). A recent study also estimated fungal contributions to ~24% of ASVs and ~14% of reads across 32 freshwater samples (Monjot et al. 2023). These comparisons reinforce the notion that fungal diversity and dominance are highest in terrestrial and freshwater habitats, declining sharply into marine environments.

The fungal community in soil samples harboured higher richness and distinct composition, as evidenced by the 18,279 OTUs found only in soil samples, representing 91.7% of all fungal OTUs. While the higher number of soil samples in our work influences this pattern (*n* = 165, against 33 in sediment and 80 in water), the higher richness in soil compared to marine environments has been found in other studies (Debeljak and Baltar 2023; Jones et al. 2014). This pattern is partly due to the higher organic carbon availability in terrestrial ecosystems (Burgaud et al. 2022). The marine community was composed mainly of Ascomycota and Basidiomycota, with a higher proportion of Chytridiomycota, Labyrinthulidia and Oomycota compared to soil, in accordance with other studies (Comeau et al. 2016; Grossart et al. 2019; Hassett et al. 2020; Picard 2017; Rojas‐Jimenez et al. 2020; Shearer et al. 2007; Wang et al. 2018).

Within the marine environment, we also observed higher richness and distinctive sediment composition compared to water samples, as indicated by the higher relative abundance of Rozellomycota (formerly Cryptomycota). Other studies have shown higher benthic richness of fungi compared to surface waters (Li et al. 2018), as demonstrated for other microbial groups such as protists (Forster et al. 2016; Massana et al. 2015) and bacteria (Zinger et al. 2011). As for soil, the higher amount of organic matter in sediment than in water could be a major factor favouring richer fungal communities. Despite the quantities of materials that could explain part of richness differences (1 L of water vs. 10 g of sediment), the distinction between water and sediment fungal composition could be attributed to various metabolic pathways (Wang et al. 2023) but also to specific fungal taxa showing biotic interactions with phytoplankton (Chrismas et al. 2023; Wang et al. 2024) or benthic organisms such as corals and sponges (Yarden 2014), as highlighted by the higher proportion of taxa annotated as animal parasites in sediment. Additionally, the presence of mangroves, especially in Dumbéa Bay, could influence the structure of our communities as mangroves are considered ‘hotspots’ for marine fungi (Arfi et al. 2012; Raghukumar 2017; Shearer et al. 2007), notably because of their influence on the composition and quantity of organic matter (Kristensen et al. 2008; Mouras et al. 2025).

At the genus level, deeper insight into potential ecological roles and interactions was possible. For instance, the high abundance of OTUs affiliated to *Exophiala* in the December water sample may reflect seasonal parasitic interactions, as this genus is known to cause diseases in various aquatic animals, including crabs (Guerra et al. 2013) and fish (Pedersen and Langvad 1989). *Penicillium* is a widespread genus, with species occurring in a large range of environments. Notably, several species are commonly found in marine ecosystems, as shown by Park et al. (2019). Some genera, such as *Lecanicillium*, appeared to be ubiquitous, occurring in both sediment and water samples despite temporal gaps between sampling collections. *Lecanicillium* includes species pathogenic to arthropods, nematodes and fungi or associated with terrestrial mammalian faeces (Su et al. 2019), the confirmed presence of such species could support transport from the watershed to the lagoon via runoff. Finally, the widespread occurrence of *Aureobasidium*, particularly *A. pullulans*, aligns with previous studies documenting its presence in various marine environments (Liu et al. 2009). The species‐level assignment using amplicon sequencing, even with long‐read technologies, may lack sufficient resolution, especially for certain taxa like *Penicillium* (Samson et al. 2004). Therefore, these ecological hypotheses require further validation through additional research.

The large proportion of unknown genera and their functional traits in marine samples compared to soil samples underlined the knowledge gaps regarding marine fungi. In his work, Picard (2017) reported a high proportion of novel sequences in the coastal marine sediment of North Carolina. Our data showed the overall presence of fungi and fungal‐like stramenopiles in all marine samples, despite not being the dominant group among all eukaryotes (in terms of read number). The ecological roles of these marine fungi are now acknowledged, such as their crucial roles in organic matter decomposition and biotic interactions, but remain unclear and warrant further investigation (Grossart et al. 2019; Krauss et al. 2011). The concept of marine fungi as ‘fungal dark matter’ pinpoints the need for more studies to understand their ecological significance (Grossart et al. 2016), particularly in underexplored tropical marine ecosystems such as New Caledonia.

### 4.2 Temporal Variability and Cyclone Impacts

As observed for protists (Caracciolo et al. 2022), planktonic fungal communities exhibit clear seasonal dynamics (Chrismas et al. 2023; Wang et al. 2018). During dry‐season sampling campaigns, coastal marine fungal diversity remained relatively low, with fewer OTUs and a taxonomic composition distinct from sediment and terrestrial sources. However, seasonal variations can significantly affect fungal taxa exchanges between terrestrial and marine areas, especially in tropical and subtropical latitudes, as shown by several studies showing the impact of flood pulse on the abundance of microbial taxa (Chen et al. 2019; Chrismas et al. 2023; Pan et al. 2022; Wang et al. 2018). This is in accordance with our results showing the influence of cyclone Uesi on fungal community composition in water samples. This extreme event, characterised by a rapid drop in salinity and a major increase in river discharge, clearly altered the environmental conditions and allowed a pulse of terrestrial fungi to enter the marine system. The temporal monitoring following the cyclone revealed the transient nature of this input: while 254 OTUs were detected on the first day, richness quickly declined over the subsequent days, stabilising to values closer to those observed during dry periods. This short‐term pattern is consistent with the ephemeral nature of some river plumes and the selective pressure exerted by a change in osmotic pressure due to a sharp increase in salinity (Mohamed and Martiny 2011; Rojas‐Jimenez et al. 2020).

Heavy runoff during wet seasons could also enhance the mixing between sediment and water communities, as observed in riverine ecosystems (Pan et al. 2022). Our results indicated that the fungal communities after the cyclone showed indeed a proportion of Ascomycota close to that in sediment, but a clearly higher proportion of Chytridiomycota and a low richness, illustrating once again the significant differences between these two compartments, even after a strong mixing event. However, the sediment and water samples were not taken at the same time period, which could also explain those differences. Nevertheless, despite this gap in the sampling period, we still evidenced 51 shared OTUs across all three sample types, highlighting a potential constant flux of fungal organisms from land to sea, regardless of temporal variations.

These findings indicate that both seasons and extreme weather events such as cyclones can act as ecological pulses, temporarily increasing the connectivity between terrestrial and marine compartments and reshaping coastal fungal communities, corroborating what was previously observed for the protistan and bacterial community (Meyneng, Lemonnier, et al. 2024). However, the rapid return to baseline fungal composition suggests strong environmental filtering at the land‐to‐sea interface. Although short‐term dispersal may be high, successful colonisation or establishment of terrestrial fungi in marine habitats appears limited, likely due to ecophysiological constraints.

### 4.3 Mechanisms of Fungal Dispersal and Persistence

Our work highlighted an overlap of fungal communities between terrestrial and marine ecosystems, providing evidence that fungi from terrestrial ecosystems can reach and transiently inhabit oligotrophic marine environments. Fungi represented by these shared OTUs might have been transported by runoff and followed the river plume through passive water dispersal. Dispersal mechanisms are often driven by external forces such as runoff, river plumes and oceanic currents (Chaudhary et al. 2022; Vellend 2010), shaping fungal community structure (Li et al. 2018). Our data showed shared fungal OTUs among soil, sediment and water samples, highlighting this connection among coastal habitats. This overlap is variable, shown by the higher number of shared OTUs between soil‐sediment (285) than soil‐water (175), despite the lower number of benthic samples, demonstrating the accumulation capacity of sediment, acting as a reservoir of terrestrial microbial inputs (Forster et al. 2016; Staley et al. 2016).

Some fungal taxa in our samples illustrated typical water‐driven dispersal, such as *Phytophthora* sp., a fungus‐like plant pathogen that disperses mainly through streams (Sutton et al. 2009), which we found only in the 2 days after the cyclone. Their presence in marine environments is associated with river transport, as ‘rivers may constitute an overlooked avenue of dispersal for terrestrial fungi’ (LeBrun et al. 2018). Some fungi are known to be adapted to aquatic transport, such as Hyphomycetes, which are mainly transported by rainwater flowing (Chauvet et al. 2016) and require specific morphological and ecophysiological properties. Dispersion by water shows some benefits for fungi compared to air dispersal, such as protection from drought or UV stress (LeBrun et al. 2018). This emphasises that dispersal can be a deterministic process, as explained by Custer et al. 2022, because some species traits, such as spore formation and their transport mechanism, can favour certain species‐dispersal and determine dispersal efficiency.

The use of the FungalTraits database (Põlme et al. 2020) allowed us to find that some of those shared OTUs correspond to taxa with known non‐aquatic lifestyles. The number of non‐aquatic OTUs was clearly higher 1 day after cyclone Uesi compared to dry seasons in water samples. Runoff events likely transport terrestrial and freshwater fungi into marine environments, enhancing fungal richness near river mouths and explaining part of the seasonal variability mentioned above (Li et al. 2018; Rojas‐Jimenez et al. 2020; Wang et al. 2018). This coalescence process and its intensity, influenced by hydrological connectivity, induce microbial community changes (Chang et al. 2024; Mansour et al. 2018). Soil erosion caused by heavy rainfall may also transport soil‐associated fungi, potentially generating spatial patterns similar to those observed in terrestrial environments of New Caledonia (Fernandez Nuñez et al. 2021; Gourmelon et al. 2016; Ripoll et al. 2025), thereby influencing coastal communities (Meyneng, Siano, et al. 2024). The samples taken during the daily monitoring after the cyclone showed less than four terrestrial OTUs after Day 2, even fewer than in the dry season (Figure 5), probably because of the complex dynamic of the area after such a massive impact (Meyneng, Lemonnier, et al. 2024). Salinity is a major factor influencing fungal communities, as highlighted by the salinity‐induced gradient from river to ocean (Burgaud et al. 2013; Mohamed and Martiny 2011; Rojas‐Jimenez et al. 2019). During the monitoring, the usual marine salinity returned to its normal value of 35 on Day 2. Given the strong contrast between terrestrial and marine conditions, particularly in salinity, many fungal OTUs may not survive this abiotic barrier. Nevertheless, we detected some rare OTUs that persisted, emphasizing the resilience of specific fungal taxa. Such resistance to salinity is a key physiological trait enabling viable dispersal, reflecting the versatility of certain fungi. Indeed, spore tolerance to marine salinity can be high for some species, with spores remaining viable for months (J. H. Anderson 1979; Koske et al. 1996). Additionally, cyclonic events may further facilitate dispersal by physically transporting plant material that serves as a vehicle for fungi. For example, (Rämä et al. 2014) found that logs sampled across the North Sea carried up to 50% non‐marine fungi, illustrating the potential for terrestrial taxa to reach marine environments via such mechanisms.

Our interpretations are further supported by previous studies conducted in temperate and subtropical coastal systems of East Asia, where fungal communities were investigated along coastal gradients. These studies consistently reported strong shifts in fungal community composition associated with salinity gradients and terrestrial inputs (e.g., Li et al. 2018; Rojas‐Jimenez et al. 2019; Wang et al. 2023). In these systems, terrestrial fungi were regularly detected in coastal waters (and sediments), but generally showed limited persistence in marine environments, suggesting strong environmental filtering despite continuous dispersal. However, these studies mainly addressed spatial or seasonal gradients under relatively stable hydrological conditions and did not capture the effects of short‐term extreme events. By contrast, our cyclone‐driven observations exemplify how extreme hydrological pulses can temporarily amplify land‐to‐sea fungal dispersal, increasing the richness of terrestrial taxa beyond levels typically observed under baseline conditions.

However, fungi use various dispersal mechanisms, and terrestrial ones in marine environments can also result from air propagation (Wyatt et al. 2013). In our data, some fungal DNA could have been dispersed by wind rather than by water. This factor cannot possibly be distinguished in our work, especially after the cyclone. A way to identify taxa that are more probably transported by water could be by focusing on root‐associated fungi, where wind transport is less probable because of their lifestyle. In our data, we recorded 15 ectomycorrhizal and six root‐endophyte taxa, and among them, three ectomycorrhizal taxa were found just after the cyclone (*Pisolithus albus*, *Rhizopogon villosulus* and *Inocybe* sp.) (Figure 5). Additionally, factors related to atmospheric dispersal, such as fire events, may explain the higher richness of terrestrial fungi observed in the marine environment during the December campaign compared to September. Fires can increase the release of fungal propagules into the atmosphere and enhance their long‐distance transport by generating strong convection and altered wind dynamics, thereby increasing deposition in marine environments (Golan and Pringle 2017). In a biodiversity hotspot like New Caledonia, identifying additional endemic indicators may be worthwhile, for instance, members of the genus *Cortinarius*, which were found to dominate specific vegetation types in the study by Ripoll et al. (2025). Nevertheless, the spores of ectomycorrhizal fungi might have been carried to the sea by wind, given their spore‐producing fruit bodies, although *Rhizopogon* is primarily dispersed by animals (Kretzer et al. 2005). The potential of these taxa to serve as bioindicators of riverine inputs remains to be confirmed, requiring further ecological insight and functional annotation. Additionally, human‐mediated dispersal represents one other factor not taken into account here, but that can have significant ecological consequences (Faulkner et al. 2024; Golan and Pringle 2017; Melnyk et al. 2023). Nonetheless, even if their presence is not directly linked to water transport, it highlights the possible dispersal of these organisms in marine areas through alternative mechanisms.

Aside from passive transport, certain fungal OTUs detected after the cyclone may have actively proliferated in response to the newly disturbed environment. For instance, an OTU assigned to *Dinomyces* sp., an algae parasite (Gleason et al. 2015), showed increased reads from the 4th day until Day 6, when the chlorophyll *a* value was about 2.5 mg L^−1^ 6 days after the input. Our results reveal a significant positive association between chytrid and diatom read numbers, suggesting that an increase in parasitic chytrid abundance might have been provoked by the increase in potential diatom hosts. This finding aligns with previous studies that report positive correlations between fungal diversity and phytoplankton biomass in coastal waters (Gao et al. 2010) and seasonal chytrid bloom related to diatom bloom (Garvetto et al. 2019; Taylor and Cunliffe 2016; Wang et al. 2023). Other taxa could be more likely transient, found the first day after the cyclone, but not recorded in the same coastal zone or the plume trajectory (buoy samples). This could illustrate an ‘unsuccessful’ dispersal event, which does not exclude ecological implications (Custer et al. 2022).

A significant proportion of taxa remained unknown in taxonomy and/or function, probably including non‐aquatic fungi that remained in coastal water after the cyclone. Despite the full‐length ITS barcoding, which allows better representation of marine‐associated fungi than the more classic ITS2 (Appendix A), a main limitation remains in the incompleteness of the taxonomic and functional database. The ongoing efforts to complete those reference databases will help us disentangle the ‘aquatic mycobiome’ with a broader context, considering the global water cycle (Grossart et al. 2019) and the connectivity among habitats highlighted in our work.

## 5 Conclusion

This study provides the first comprehensive description of fungi and fungal‐like stramenopile communities along a tropical land‐to‐sea continuum, combining long‐read metabarcoding with ecological trait annotation across soil, sediment and surface water samples. Our findings highlight the episodic nature of microbial dispersal at the land–to‐sea interface and the influence of hydrological pulses in shaping coastal mycobiomes. Although the application of fungi in environmental monitoring is currently constrained by airborne dispersal and gaps in taxonomic and functional resolution, our results indicate, as hypothesised, that terrestrial fungi have clear potential as indicators of land‐derived inputs. Such indicators may be especially informative when fungal signatures persist longer than associated water masses, and the benthic reservoir represents a valuable target for assessing the spatial extent of dispersal. Overall, elucidating fungal connectivity across ecosystems can enhance long‐term biomonitoring, refine ecological models of dispersal and connectivity, and ultimately improve our capacity to anticipate ecosystem responses under accelerating global change.

## Author Contributions

**M. Meyneng:** conceptualization, software, formal analysis, visualization, writing – original draft, writing – review and editing. **L. Tedersoo:** conceptualization, resources, supervision, writing – review and editing. **G. Burgaud:** writing – review and editing. **V. Mikryukov:** writing – review and editing, resources, software. **F. Carriconde:** writing – review and editing. **H. Lemonnier:** conceptualisation, writing – review and editing. **R. Siano:** conceptualization, resources, supervision, writing – review and editing.

## Funding

This work was supported by Institut Français de Recherche pour l'Exploitation de la Mer.

ISblue (ANR‐17‐EURE‐0015).

## Conflicts of Interest

The authors declare no conflicts of interest.

## Supporting information


**Data S1:** emi470297‐sup‐0001‐Data S1.docx.

**Data S2:** Table of the quantification of sample sequencing completeness.

**Data S3:** Table of the 445 genera/species with their functional annotations (habitat and primary lifestyle).

## Figures and Tables

**FIGURE A1:**
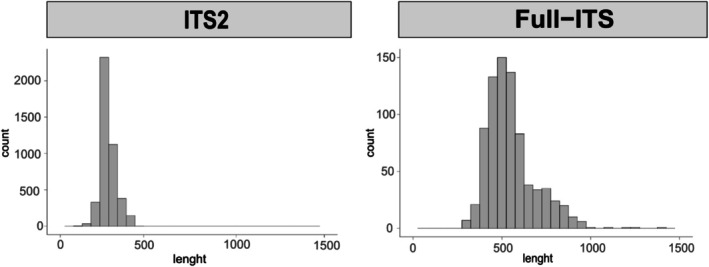
Length of the amplicon of both markers.

**FIGURE A2:**
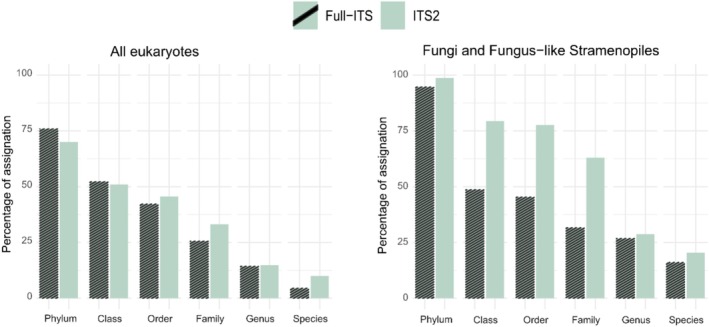
Percentage of assignments at different taxonomic levels depending on the marker used.

**FIGURE A3:**
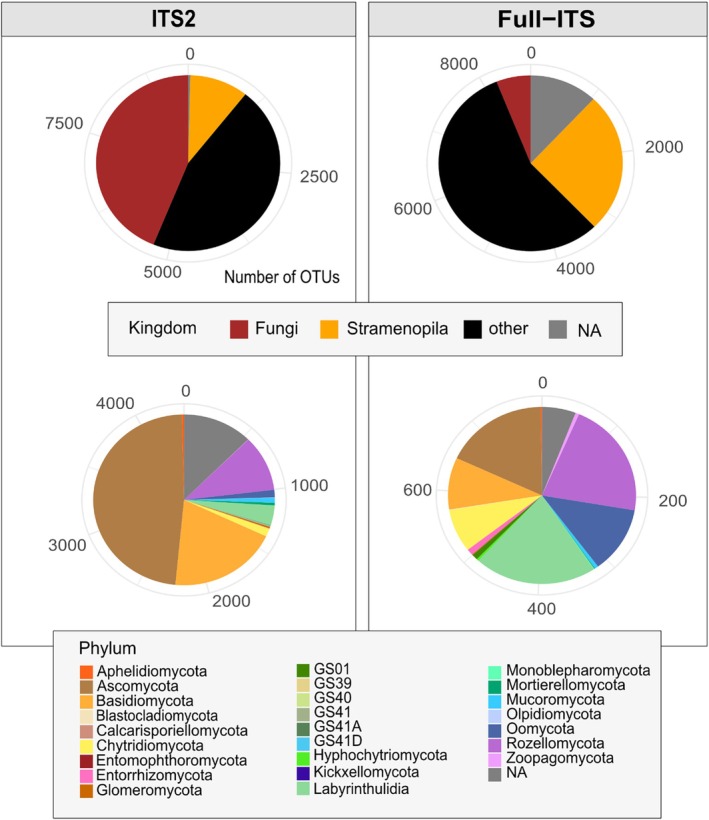
Differences in taxonomic composition between ITS2 and Full‐ITS at the kingdom level for all eukaryotes and phylum levels for fungi and fungus‐like stramenopiles.

## Data Availability

The data that support the findings of this study are openly available in PRJNA1294814 at https://www.ncbi.nlm.nih.gov/bioproject/PRJNA1294814/.

## Appendix A Taxonomic Resolution and Community Composition Depending on Amplicon Sequencing Methods

### Methods for Amplicon Sequencing of ITS2 Region

The ITS2 region was sequenced on a subset of 26 samples (22 sediment and 4 water samples) to compare with the sequencing of the full‐length ITS region. The amplification was done using gITS7ngs/ITS4ngsUni primers. PCR was done using the HOT FIREPol Blend Master Mix (Solis Biodyne, Tartu, Estonia). PCR program was composed of an initial denaturation at 95°C for 15 min; 38 cycles of denaturation at 95°C for 30 s, annealing at 55°C for 30 s and elongation at 72°C for 1 min; final elongation at 72°C for 10 min. Positive and negative controls followed the same process to limit contamination bias. Similarly to the full ITS protocol, amplification was verified by gel electrophoresis, and the library was prepared and purified. The ITS2 library was sequenced with an Illumina MiSeq.

### Results

The length of the amplified fragments varied, with the PacBio method producing longer and more variable length fragments (Figure A1). Both markers provided relatively similar taxonomic resolution at the genus and species levels, which are of primary interest here (Figure A2). At the class, order, and family level, the ITS2 marker showed a higher percentage of taxonomic assignment. Thus, this variation in assignment percentages was strongly influenced by the composition detected by each marker. The ITS2 marker predominantly detected Ascomycota and Basidiomycota. In contrast, the full‐ITS marker identified a higher proportion of taxa linked to marine environments, such as Chytridiomycota, Labyrinthulomycetes, Rozellomycota and Oomycota (Figure A3). The higher representation of these lesser‐known and less well‐annotated marine taxa with the full‐ITS marker explained the low percentage of assignment, despite the use of a longer marker supposed to improve taxonomic resolution.
