# Supplementary material for: Diversity and Dispersal of Fungi Along a Subtropical Land‐to‐Sea Continuum
Source: Environ Microbiol Rep. 2026 Apr 3;18(2):e70297. doi: 10.1111/1758-2229.70297 (PMC13052330; doi:10.1111/1758-2229.70297)
Supplement: Supplementary file 1 — Data S1: emi470297‐sup‐0001‐Data S1.docx. [file EMI4-18-e70297-s001.docx]

Diversity and dispersal of fungi along a subtropical land-to-sea continuum

M. Meyneng, L. Tedersoo, G. Burgaud, V. Mikryukov, F. Carriconde, H. Lemonnier, R. Siano

**Supplementary material 1**

Table of contents

**Table S1**: Value of temperature, salinity, and chlorophyll *a* in marine samples.

**Figure S1**: Impact of sequencing read depth on the number of identified OTUs in different substrates. OTU accumulation curves are shown for individual samples.

**Figure S2**: Length of amplified sequences.

**Figure S3**: Percentage representation of fungi and fungal-like stramenopiles among all OTUs and reads in soil, sediment, and water samples.

**Figure S4**: Number of fungal OTUs per sample among the different substrates.

**Figure S5**: Beta-diversity of fungal community among marine samples (sediment and water) at different taxonomic levels.

**Figure S6**: Phylum composition of fungal communities detailed in all soil samples.

**Figure S7**: Variability in fungal community richness, expressed as the Shannon diversity index and OTU richness, in sediment and water samples.

**Figure S8**: Variability in the number of fungal taxa assigned to aquatic and terrestrial in sediment and water samples.

**Figure S9**: Relationship between Chytridiomycota and diatoms (Bacillariophyta) in the water and sediment samples

***Table S1****: Value of temperature, salinity, and chlorophyll a in marine samples. (* in µg/L for water samples and µg/g of wet sediment for sediment samples)*

| Sample | Group | Matrix | Date | Temperature (°C) | Salinity | Chl a (*) |
| --- | --- | --- | --- | --- | --- | --- |
| sept-A | Sept-Coast | Water_dry_season | 23/09/2019 | 22.5 | 35.4 | 0.6 |
| sept-B | Sept-Inter | Water_dry_season | 23/09/2019 | 22.3 | 35.6 | 0.2 |
| sept-C | Sept-Off | Water_dry_season | 23/09/2019 | 22.2 | 35.7 | 0.2 |
| sept-D | Sept-Coast | Water_dry_season | 24/09/2019 | 22.6 | 35.6 | 0.4 |
| sept-G | Sept-Coast | Water_dry_season | 25/09/2019 | 22.2 | 35.5 | 0.4 |
| sept-H | Sept-Inter | Water_dry_season | 25/09/2019 | 22.3 | 35.7 | 0.2 |
| sept-I | Sept-Off | Water_dry_season | 25/09/2019 | 22.3 | 35.6 | 0.2 |
| dec-A | Dec-Coast | Water_dry_season | 08/12/2020 | 26.6 | 35.9 | 0.7 |
| dec-B | Dec-Inter | Water_dry_season | 08/12/2020 | 26.0 | 35.9 | 0.3 |
| dec-C | Dec-Off | Water_dry_season | 08/12/2020 | 25.7 | 35.8 | 0.2 |
| dec-D | Dec-Coast | Water_dry_season | 10/12/2020 | 25.8 | 35.7 | 0.3 |
| dec-G | Dec-Coast | Water_dry_season | 09/12/2020 | 25.3 | 35.8 | 0.7 |
| dec-H | Dec-Inter | Water_dry_season | 09/12/2020 | 26.0 | 35.8 | 0.2 |
| dec-I | Dec-Off | Water_dry_season | 09/12/2020 | 25.3 | 35.7 | 0.2 |
| DB-D1 | DB1 | Water_after_cyclone | 12/02/2020 | 23.9 | 2.2 | 0.7 |
| DB-D2 | DB2 | Water_after_cyclone | 13/02/2020 | 27.7 | 35.9 | 1.0 |
| DB-D3 | DB3 | Water_after_cyclone | 14/02/2020 | 28.7 | 35.1 | 1.3 |
| DB-D4 | DB4 | Water_after_cyclone | 15/02/2020 | 29.8 | 33.1 | 0.8 |
| DB-D5 | DB5 | Water_after_cyclone | 16/02/2020 | 28.3 | 35.6 | 1.8 |
| DB-D6 | DB6 | Water_after_cyclone | 17/02/2020 | 28.0 | 34.7 | 2.5 |
| B1-D2 | Buoy1 | Water_after_cyclone | 13/02/2020 | 26.9 | 35.4 | 0.6 |
| B1-D3 | Buoy2 | Water_after_cyclone | 14/02/2020 | 27.6 | 35.1 | 0.8 |
| B2-D2 | Buoy2 | Water_after_cyclone | 14/02/2020 | 27.6 | 35.4 | 0.5 |
| B2-D3 | Buoy2 | Water_after_cyclone | 15/02/2020 | 27.8 | 35.5 | 0.4 |
| DB-a1 | DB-a | Sediment | 23/03/2022 | 28.7 | 35.0 | 276 |
| DB-a2 | DB-a | Sediment | 23/03/2022 | 28.7 | 35.0 | 1794 |
| DB-a3 | DB-a | Sediment | 23/03/2022 | 28.7 | 36.0 | 1200 |
| DB-b1 | DB-b | Sediment | 23/03/2022 | 29.2 | 34.0 | 1426 |
| DB-b2 | DB-b | Sediment | 23/03/2022 | 29.2 | 35.0 | 1036 |
| DB-b3 | DB-b | Sediment | 23/03/2022 | 29.2 | 35.0 | 1459 |
| DB-c1 | DB-c | Sediment | 23/03/2022 | 28.9 | 36.0 | 761 |
| DB-c2 | DB-c | Sediment | 23/03/2022 | 28.9 | 32.0 | 526 |
| DB-c3 | DB-c | Sediment | 23/03/2022 | 28.9 | 32.0 | 749 |
| DB-d1 | DB-d | Sediment | 24/03/2022 | 28.5 | 35.0 | 805 |
| DB-d2 | DB-d | Sediment | 24/03/2022 | 28.5 | 37.0 | 1005 |
| DB-d3 | DB-d | Sediment | 24/03/2022 | 28.5 | 35.0 | 734 |
| DB-e1 | DB-e | Sediment | 24/03/2022 | 28.6 | 35.0 | 1334 |
| DB-e2 | DB-e | Sediment | 24/03/2022 | 28.6 | 35.0 | 1014 |
| DB-e3 | DB-e | Sediment | 24/03/2022 | 28.6 | 35.0 | 1896 |
| Co-a1 | Co-a | Sediment | 12/04/2022 | 26.0 | 35.0 | 881 |
| Co-a2 | Co-a | Sediment | 12/04/2022 | 26.0 | 36.0 | 484 |
| Co-a3 | Co-a | Sediment | 12/04/2022 | 26.0 | 35.0 | 614 |
| Co-b1 | Co-b | Sediment | 12/04/2022 | 28.5 | 35.0 | 1919 |
| Co-b2 | Co-b | Sediment | 12/04/2022 | 28.5 | 28.0 | 1408 |
| Co-b3 | Co-b | Sediment | 12/04/2022 | 28.5 | 36.5 | 1312 |
| Co-c1 | Co-c | Sediment | 12/04/2022 | 27.7 | 36.0 | 393 |
| Co-c2 | Co-c | Sediment | 12/04/2022 | 27.7 | 36.0 | 496 |
| Co-c3 | Co-c | Sediment | 12/04/2022 | 27.7 | 36.0 | 570 |
| PI-a1 | Pi-a | Sediment | 13/04/2022 | 26.5 | 35.0 | 1517 |
| PI-a2 | Pi-a | Sediment | 13/04/2022 | 26.5 | 27.5 | 515 |
| PI-a3 | Pi-a | Sediment | 13/04/2022 | 26.5 | 35.0 | 2469 |
| PI-b1 | Pi-b | Sediment | 13/04/2022 | 28.9 | 27.0 | 1470 |
| PI-b2 | Pi-b | Sediment | 13/04/2022 | 28.9 | 33.0 | 3274 |
| PI-b3 | Pi-b | Sediment | 13/04/2022 | 28.9 | 35.0 | 3912 |
| PI-c1 | Pi-c | Sediment | 13/04/2022 | 29.0 | 27.0 | 2916 |
| PI-c2 | Pi-c | Sediment | 13/04/2022 | 29.0 | 25.0 | 3264 |
| PI-c3 | Pi-c | Sediment | 13/04/2022 | 29.0 | 25.0 | 3242 |


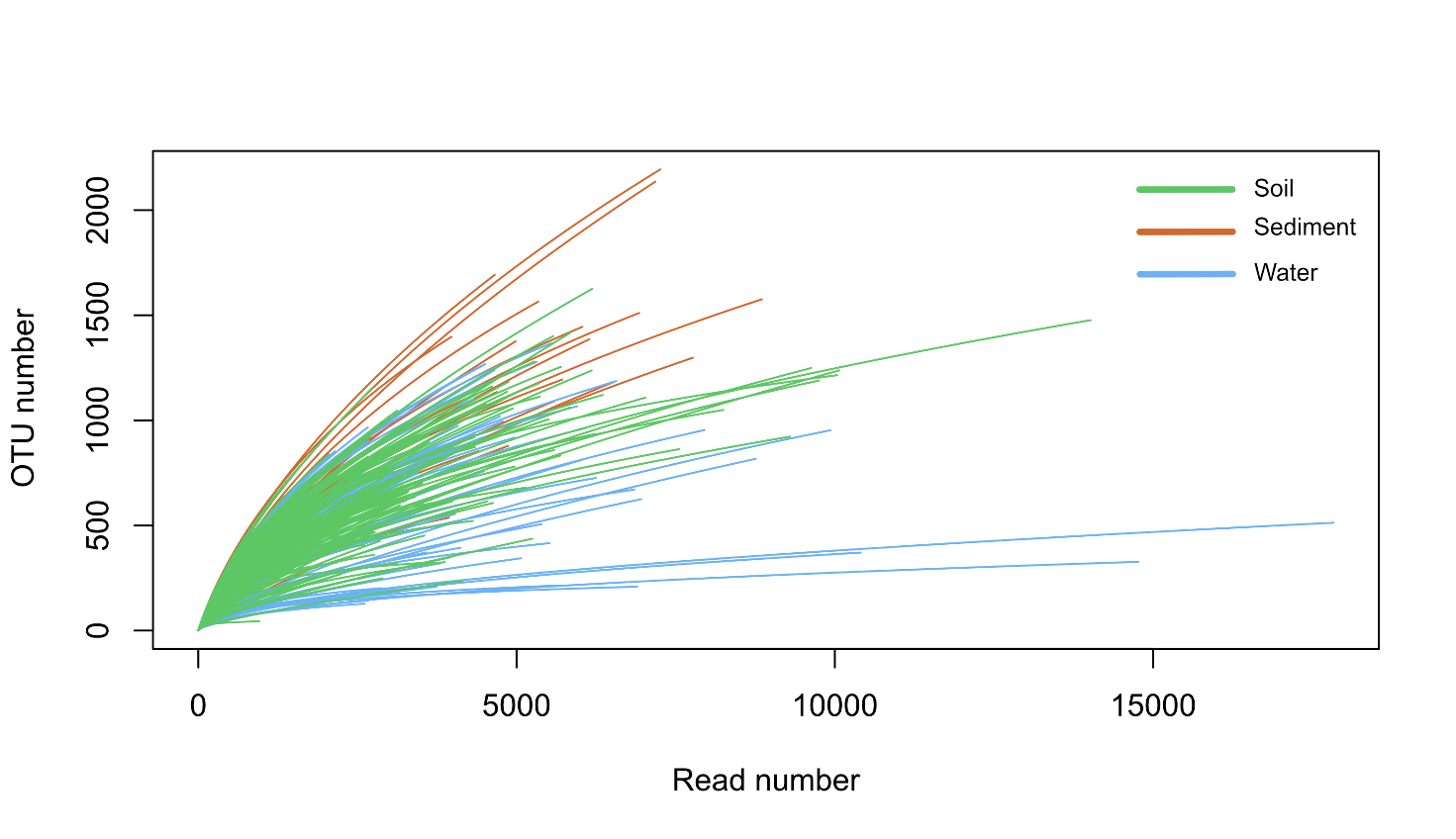


***Figure S1****: Impact of sequencing read depth on the number of identified OTUs in different substrates. OTU accumulation curves are shown for individual samples.*


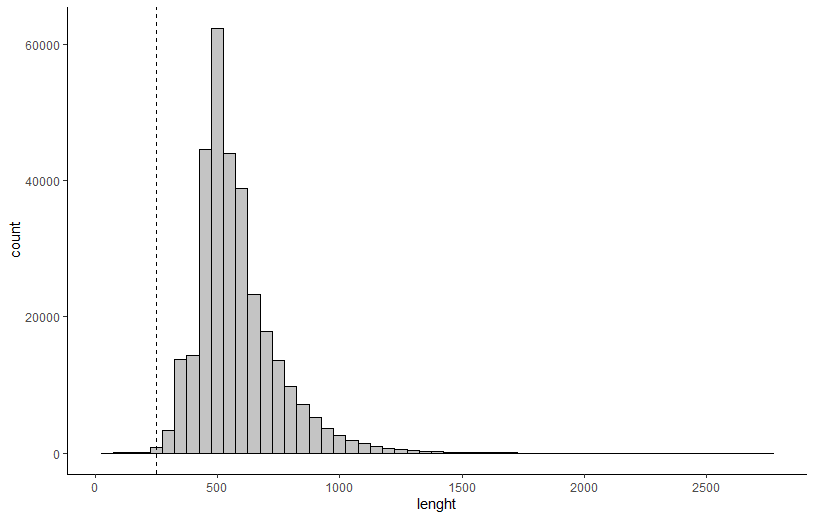


***Figure S2****: Length distribution of amplified sequences. The dotted line indicates the 250 bp cut-off threshold.*


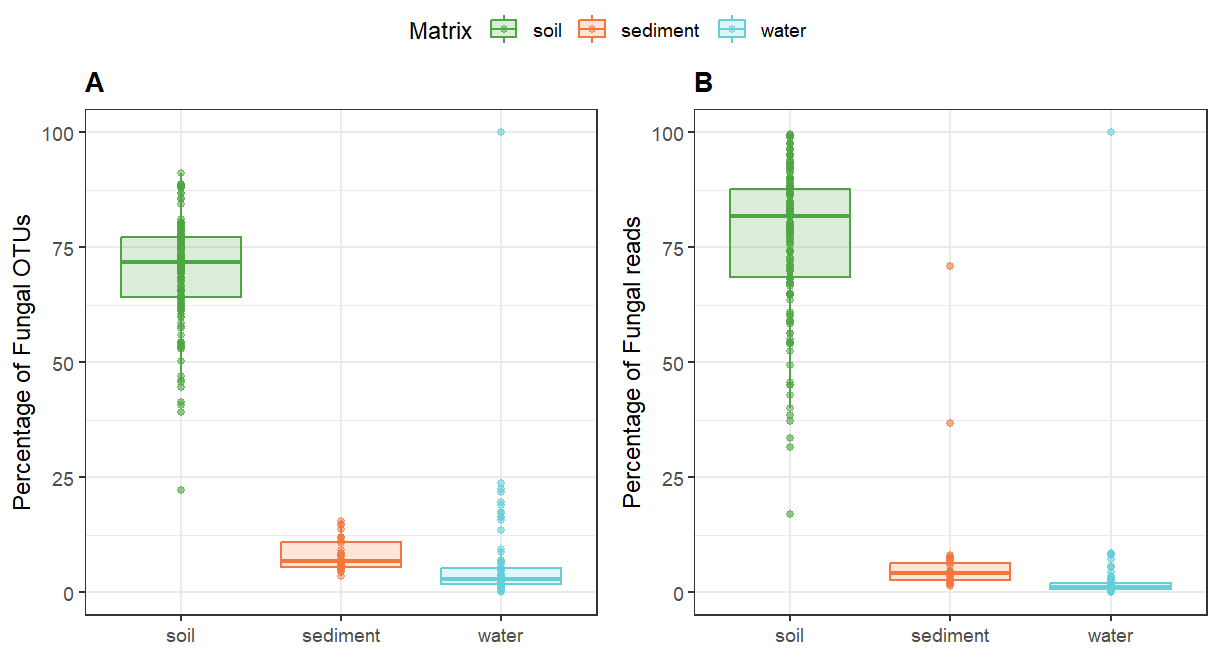


***Figure S3****: Contribution of fungi and fungal-like stramenopiles among all microeukaryote OTUs (A) and reads (B) in soil, sediment, and water samples.* *Significant differences were detected among substrates for both OTUs and reads (Kruskal–Wallis, p < 0.01). Post-hoc Dunn tests showed significant pairwise differences between soil and sediment (p < 0.01) and between soil and water (p < 0.01) for both OTUs and reads. A significant difference between sediment and water was detected only for reads (p < 0.05, p = 0.025), whereas the OTU-based comparison was marginal (p = 0.06).*


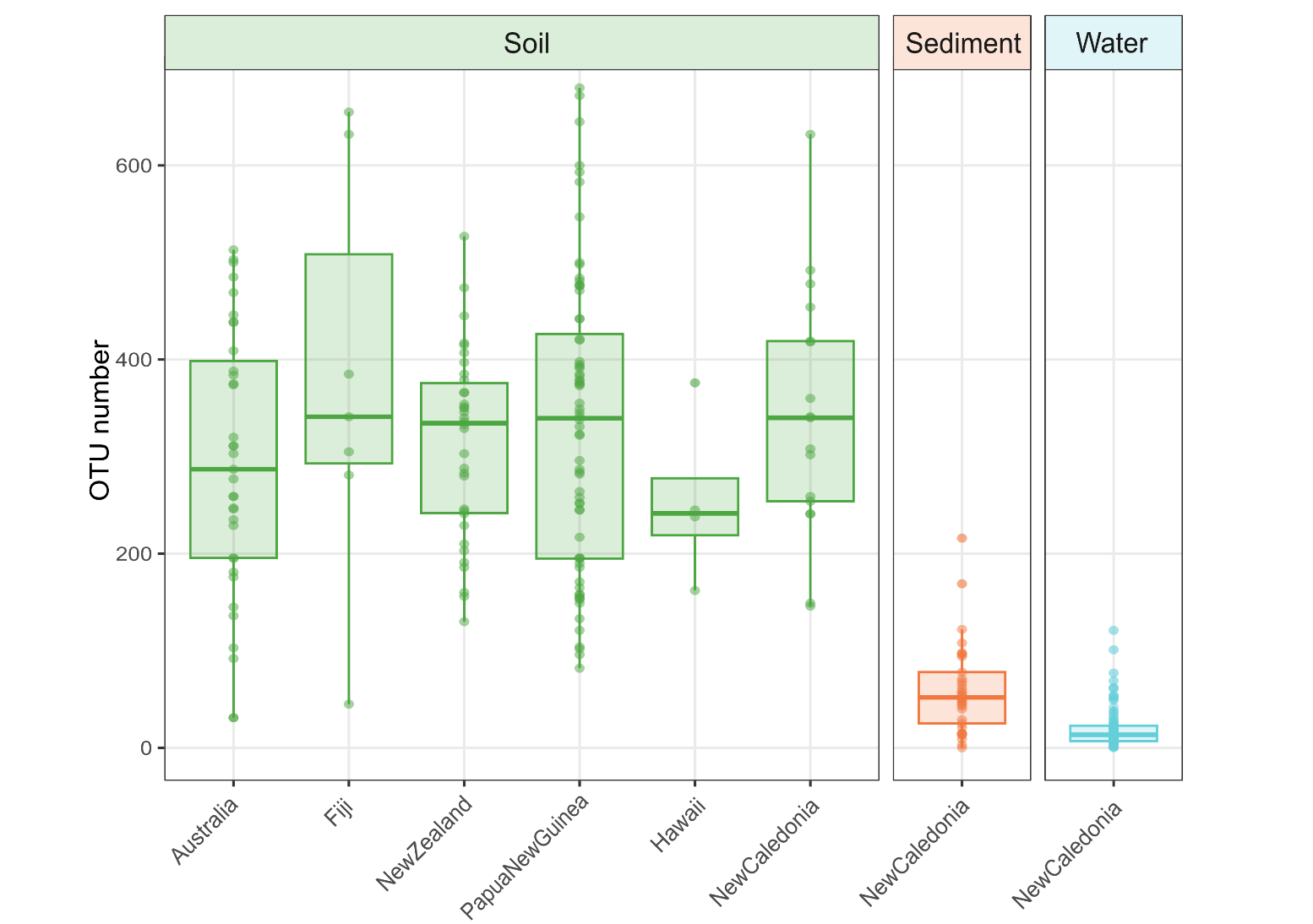


***Figure S4****: Number of fungal OTUs per sample across the three substrates.* *Significant differences were detected among substrates (Kruskal–Wallis, p < 0.01). Post-hoc Dunn tests revealed pairwise differences between soil and sediment (p < 0.01), soil and water (p < 0.01), and between sediment and water (p < 0.05, p = 0.025).*


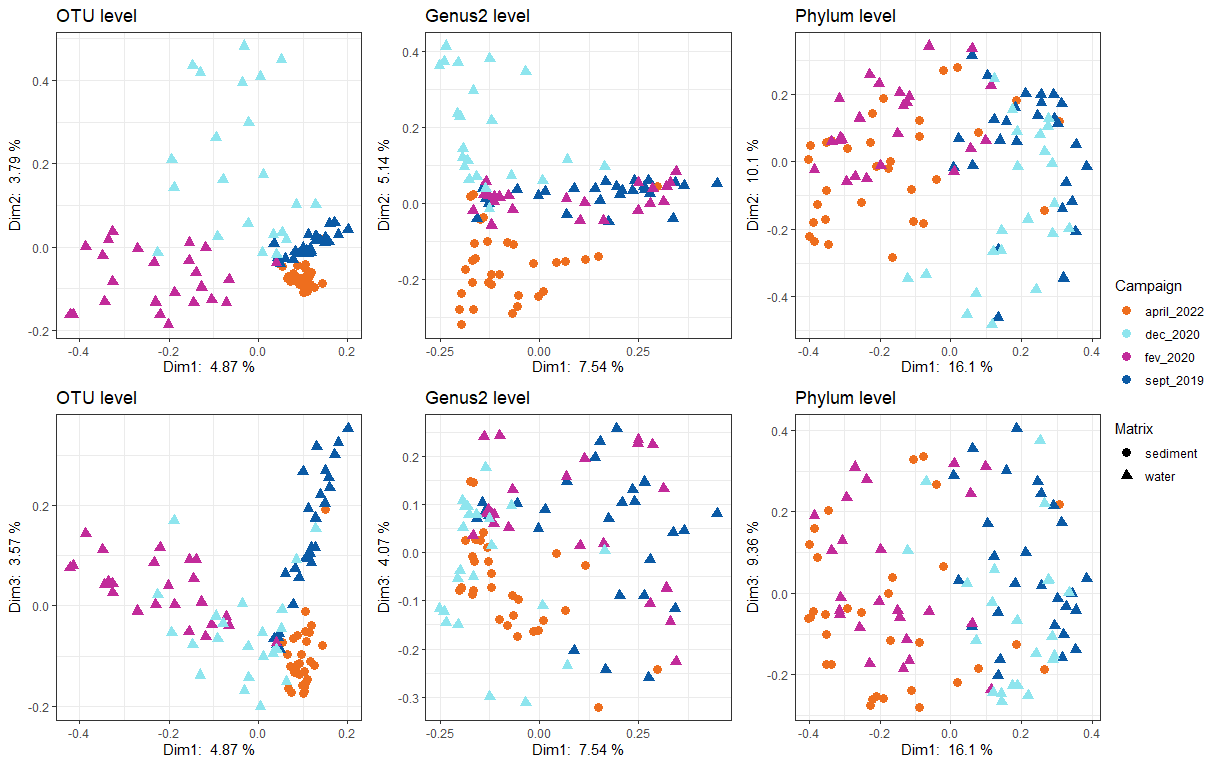
***Figure S5****: Beta-diversity of fungal community among marine samples (sediment and water) at different taxonomic levels visualised with PCoA based on Jaccard dissimilarity. The first row shows the visualisation on dimensions 1 and 2, and the second row shows the visualisation on dimensions 1 and 3.*


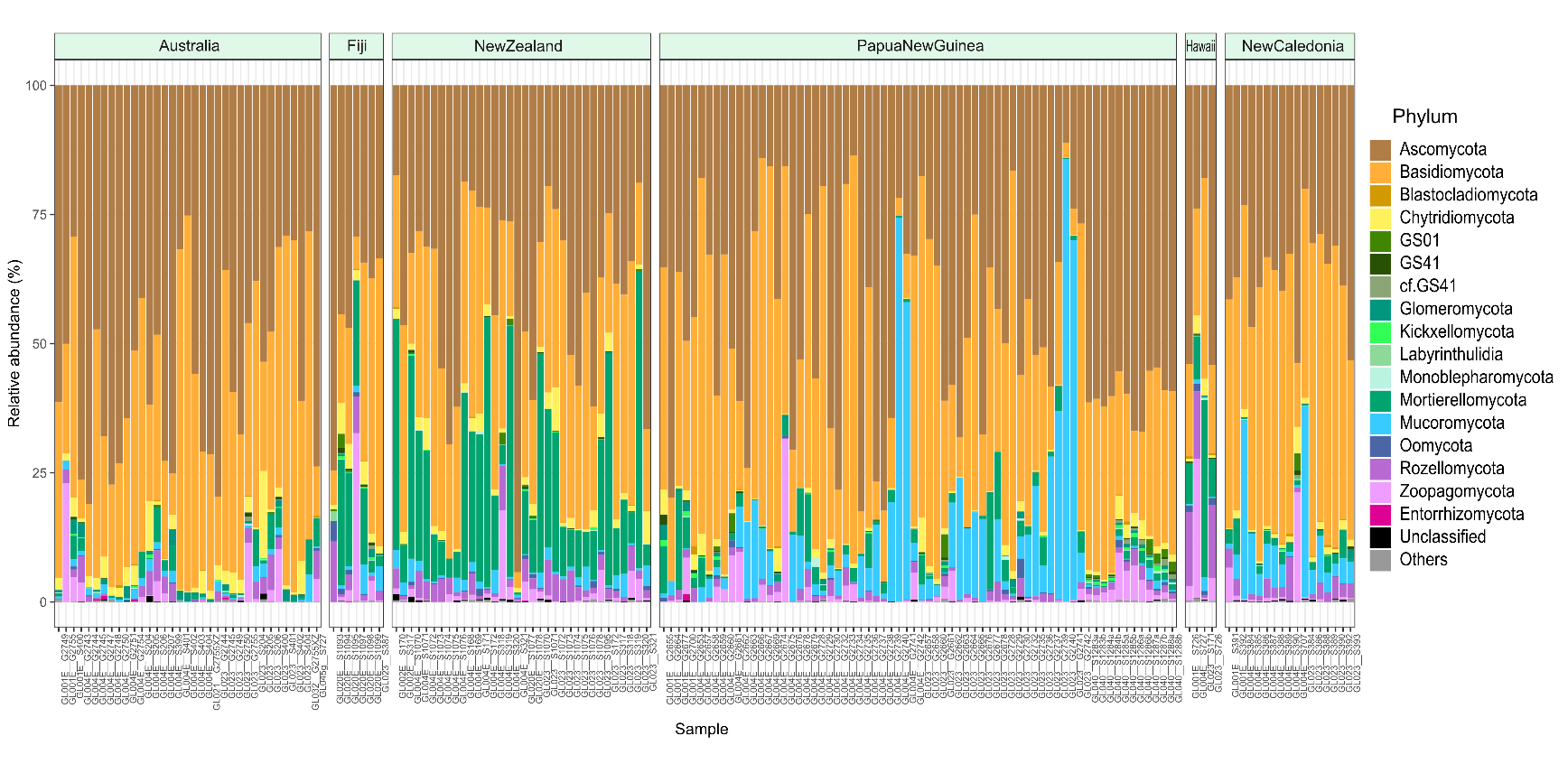


***Figure S******6****: Phylum composition of fungal communities in soil samples.*


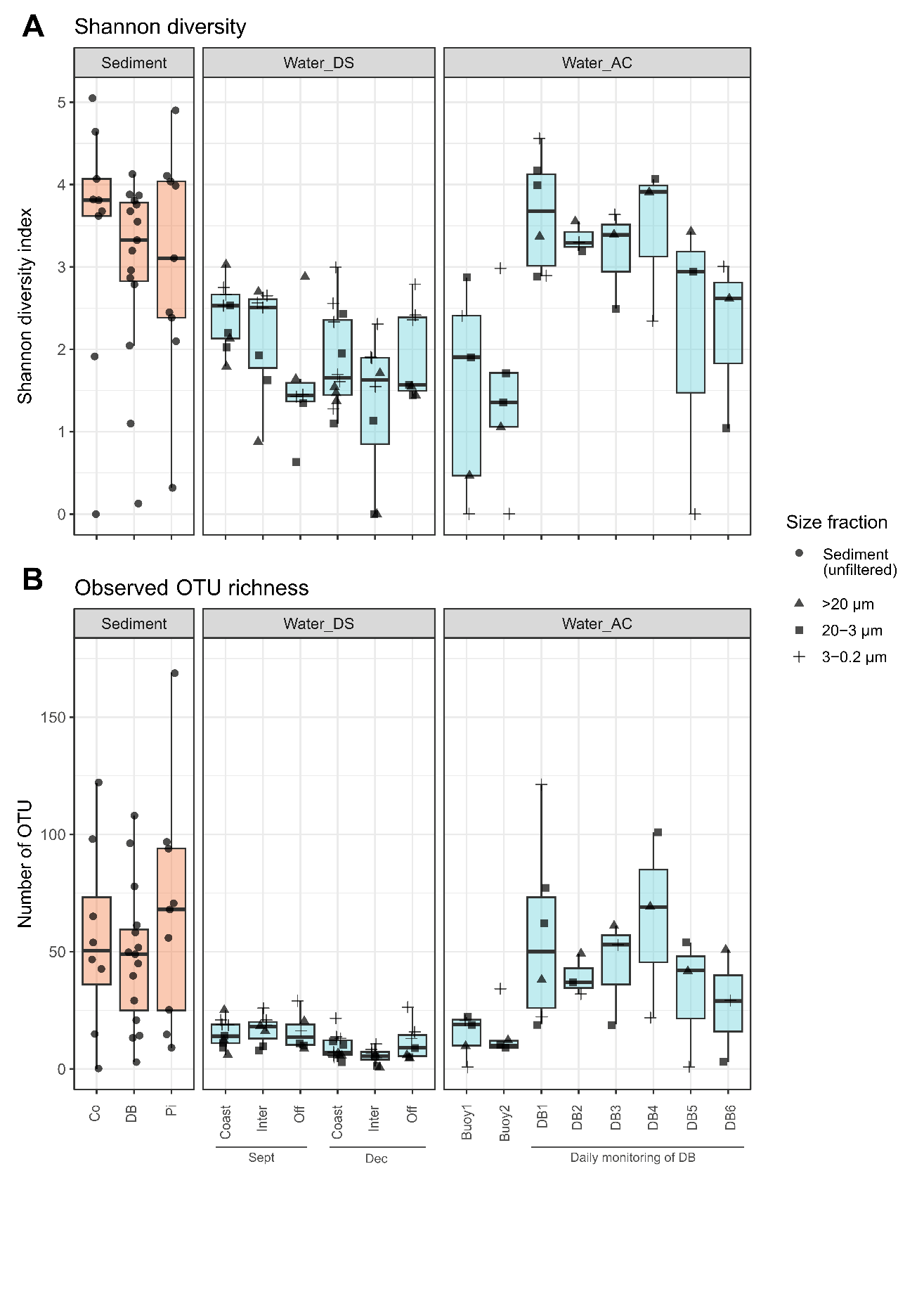


***Figure S7****: Variability in fungal community richness, expressed as the Shannon diversity index and OTU richness, in sediment and water samples. Significant differences were detected among substrates and sampling periods for both the Shannon and Observed indices (Kruskal–Wallis test, p < 0.01). Post hoc Dunn tests revealed significant pairwise differences between sediment and water during the dry season (p < 0.01), as well as between the dry season and the post-cyclone period (p < 0.01) for both indices. No significant differences were observed between sediment and water after the cyclone (Shannon: p = 0.078; Observed: p = 0.056).*

*
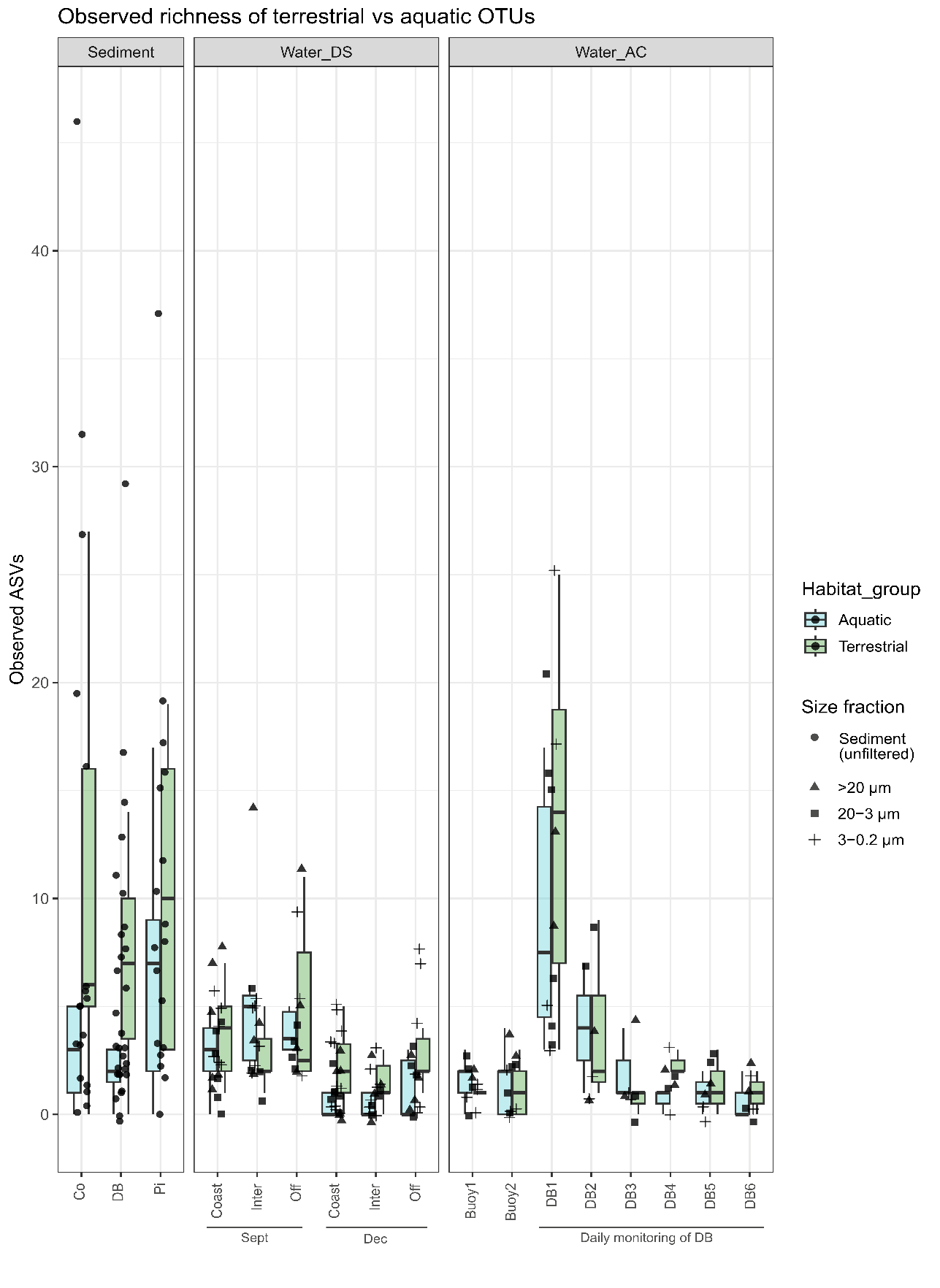
****Figure S8****: Variability in the number of fungal taxa assigned to aquatic (1 OTU “aquatic”, 48 OTUs “marine”, and 179 OTUs “partly_aquatic”) and terrestrial (306 OTUs) in sediment and water samples. Significant differences were detected among substrates and sampling periods for both aquatic and terrestrial taxa (Kruskal–Wallis test, p < 0.01). Similarly, for both groups of taxa, post hoc Dunn tests revealed significant pairwise differences only between sediment and water samples (p < 0.01). No significant differences were observed between water samples from dry seasons and after the cyclone (Aquatic: p = 0.317; Terrestrial: p = 0.320).*


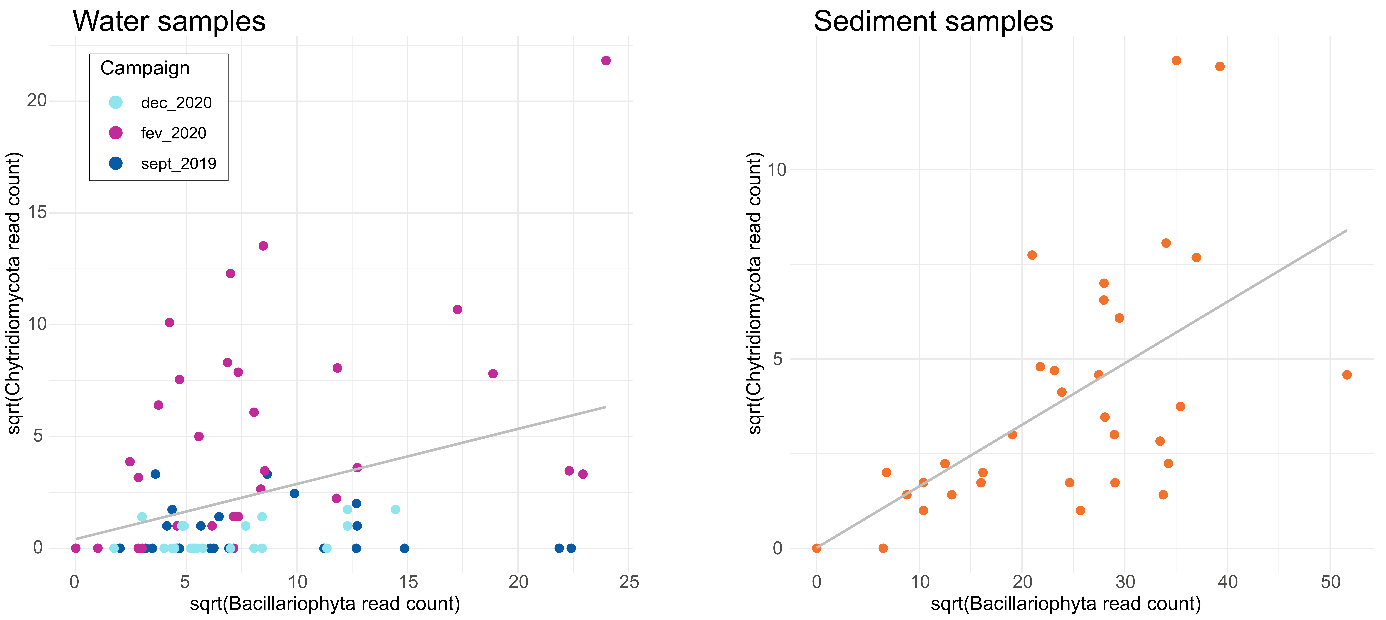


***Figure S9****: Relationship between Chytridiomycota and diatoms (Bacillariophyta) in the water and sediment samples (water samples: R^2^adj: 0.11, p-value < 0.005; sediment samples: R^2^adj: 0.31, p-value < 0.001).*
